# Guest-protein incorporation into solvent channels of a protein host crystal (hostal)

**DOI:** 10.1107/S2059798321001078

**Published:** 2021-03-30

**Authors:** Janina Sprenger, Jannette Carey, Alexander Schulz, Fleur Drouard, Catherine L. Lawson, Claes von Wachenfeldt, Sara Linse, Leila Lo Leggio

**Affiliations:** a Department of Chemistry, University of Copenhagen, DK-2100 Copenhagen, Denmark; b Center for Molecular Protein Science, Lund University, SE-221 00 Lund, Sweden; c Deutsches Elektronen-Synchrotron DESY, Notkestrasse 85, D-22607 Hamburg, Germany; d Chemistry Department, Princeton University, Princeton, NJ 08544, USA; e Department of Plant and Environmental Sciences, University of Copenhagen, DK-1871 Frederiksberg, Denmark; f Institute for Quantitative Biomedicine, Rutgers University, Piscataway, NJ 08854, USA; g Department of Biology, Lund University, SE-221 00 Lund, Sweden

**Keywords:** host–guest system, diffusion, mesopores, solvent channels, *MOLEonline*, protein volume fraction, encapsulation, hostals

## Abstract

Soaking small molecules into the solvent channels of protein crystals is the most common method of obtaining crystalline complexes with ligands such as substrates or inhibitors. The solvent channels of some protein crystals are large enough to allow the incorporation of macromolecules, but soaking of protein guests into protein crystals has not been reported. Such protein host crystals (here given the name hostals) incorporating guest proteins may be useful for a wide range of applications in biotechnology, for example as cargo systems or for diffraction studies analogous to the crystal sponge method. The present study takes advantage of crystals of the *Escherichia coli* tryptophan repressor protein (ds-TrpR) that are extensively domain-swapped and suitable for incorporating guest proteins by diffusion, as they are robust and have large solvent channels. Confocal fluorescence microscopy is used to follow the migration of cytochrome *c* and fluorophore-labeled calmodulin into the solvent channels of ds-TrpR crystals. The guest proteins become uniformly distributed in the crystal within weeks and enriched within the solvent channels. X-ray diffraction studies on host crystals with high concentrations of incorporated guests demonstrate that diffraction limits of ∼2.5 Å can still be achieved. Weak electron density is observed in the solvent channels, but the guest-protein structures could not be determined by conventional crystallographic methods. Additional approaches that increase the ordering of guests in the host crystal are discussed that may support protein structure determination using the hostal system in the future. This host system may also be useful for biotechnological applications where crystallographic order of the guest is not required.

## Abstract 2

This study reports the incorporation of small guest proteins by diffusion (soaking) into the solvent channels of a host protein crystal, visualized in time and space by microscopic techniques. The results represent a first step towards the realization of a protein-based crystal host (‘hostal’) system that may be used in the future as a carrier/entrainment system for protein guests and/or for guest-protein structure determination.

## 1. Introduction   

Many areas of life science rely on structural information about proteins, for example to provide a basis for the design of experiments to elucidate biochemical processes in cells, to tailor industrial biocatalysts or to support computer-aided drug design (Shi, 2014 ▸; Blundell, 2017 ▸). The majority of high-resolution structures of proteins in the Protein Data Bank (PDB; http://www.rcsb.org/stats/summary) have been determined by X-ray crystallographic methods, and this continues to be the case despite recent advances in solution and solid-state NMR spectroscopy as well as cryo-electron microscopy. However, protein crystals that diffract X-rays are not always easy to obtain, partly because, unlike crystals of small molecules and compounds, they typically contain large amounts of solvent: between ∼25% and ∼75% of the total crystal volume (Matthews, 1968 ▸), with a mean of ∼50% (Weichenberger & Rupp, 2014 ▸). Solvent is present throughout protein crystals in a network of channels that can be organized as micropores or mesopores (Falkner *et al.*, 2005 ▸). The continuity of solvent in the channels can be exploited by soaking crystals in solutions containing protein ligands, drug candidates or other compounds of interest (McNae *et al.*, 2005 ▸). Such soaking experiments typically use molecules that are much smaller than the crystallized proteins. Many ligands reach their binding sites within seconds to minutes (Mizutani *et al.*, 2014 ▸; Collins *et al.*, 2017 ▸), but the binding-site occupancy may still increase after hours of soaking (Collins *et al.*, 2017 ▸). To follow the soaking processes of ligands into crystals experimentally, X-ray diffraction data can be used to calculate ligand occupancies as a function of soaking time (Cole *et al.*, 2017 ▸) for ligands that attain sufficient order to be structurally analyzed. Drug fragment-screening approaches have made use of soaking as a high-throughput method to introduce small-molecule ligands from chemical libraries into crystals of a protein drug target, with analysis by X-ray diffraction (Murray & Rees, 2009 ▸). Soaking of small fluorescent molecules into protein crystals can also be followed using confocal laser-scanning microscopy (CLSM), as described for lysozyme crystals (Cvetkovic *et al.*, 2004 ▸, 2005 ▸), and is not limited to molecules that adopt crystallographic order.

Despite the enormous success of protein crystallography, obtaining crystals of a protein of interest is still a major bottleneck in protein structure determination and can be very time- and resource-consuming, or even unsuccessful for some proteins (Giegé, 2017 ▸). The use of so-called crystallization chaperones, including antibodies and designed ankyrin-repeat proteins (DARPins), has resulted in several new crystal structures (Benvenuti & Mangani, 2007 ▸), but does not bypass the search for crystallization conditions. In the last decade, a soaking technique has been developed to allow X-ray diffraction-based structure determination of small-molecule guests in crystals of unrelated small-molecule hosts, which removes the need for crystallization of the guest. In this so-called crystal sponge method, guest molecules are incorporated via diffusion into the solvent channels of an existing host crystal, often a metal–organic framework, although metal-free porous crystals have also been used (Inokuma *et al.*, 2013 ▸; Sanna *et al.*, 2015 ▸; Hoshino *et al.*, 2016 ▸). Optimization of this method (Hoshino *et al.*, 2016 ▸) has allowed the structure determination of difficult cases, including guest compounds with axial or planar chirality (Yoshioka *et al.*, 2015 ▸), small flexible compounds (Ning *et al.*, 2016 ▸) and modified metabolites (Rosenberger *et al.*, 2020 ▸) or other guest compounds in aqueous solution (Poel *et al.*, 2019 ▸). The narrow solvent channels of most organic and metal-organic frameworks limit this technique to small-molecule guests.

In parallel, various encapsulation systems for proteins have been explored for a wide range of applications. These include virus capsids (Minten *et al.*, 2009 ▸), other protein cages (Azuma *et al.*, 2016 ▸) and hydrogels (Shimanovich *et al.*, 2015 ▸), as well as DNA origami networks that were adapted to incorporate guest peptides using specific peptide–DNA interactions (Sprengel *et al.*, 2017 ▸). Applications include slow-release delivery systems, mini-bioreactors, the expression of toxic proteins and structural analysis. A crystal-based system for protein encapsulation has been reported (Fujita *et al.*, 2012 ▸) that uses a self-assembling metal–organic host designed to encapsulate the small protein ubiquitin covalently bound to building blocks that form a crystalline cage containing the attached protein guest. Even though this system is based on a host that forms crystals, only limited structural information could be recovered from X-ray diffraction data using the maximum-entropy method (Fujita *et al.*, 2012 ▸).

**Figure 1:**
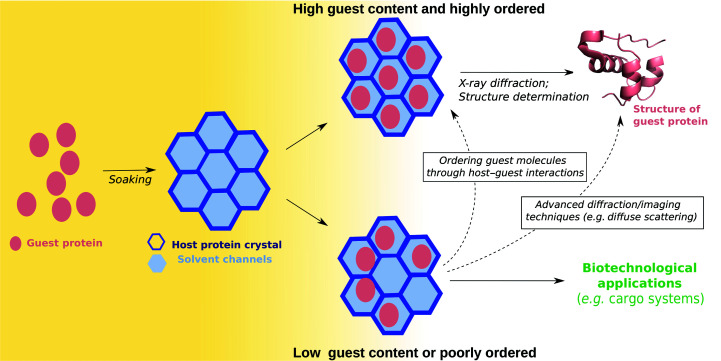
Hostal method. A guest protein (pink ovals) is introduced by diffusion (soaking) into a protein host crystal (dark blue hexagonal outlines), where it occupies the solvent channels (light blue filled hexagons). If guest-protein molecules populate the solvent channels to a sufficient extent and with high order, the guest-protein structure can in principle be solved using conventional structure-determination methods (upper path). If guest-protein molecules populate the solvent channels incompletely or with lower order (lower path), then recovering structural information for the guest protein is not trivial and requires future work, as indicated by the dotted arrows and text boxes. Non-ordered guest incorporation may be further explored for biotechnological applications (green). The present study investigates the incorporation of protein guests into a host crystal via soaking (yellow shading on the left).

**Figure 2:**
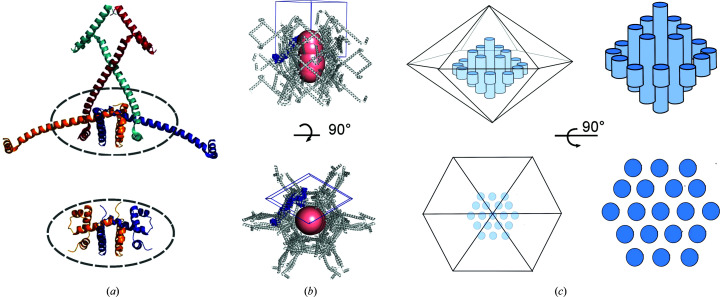
Crystals of TrpR. (*a*) Domain-swapped (top; PDB entry 1mi7; Lawson *et al.*, 2004 ▸) and dimeric (bottom; PDB entry 1jhg; Lawson, 1996 ▸) structures of TrpR in a schematic ribbon view. The four polypeptide chains comprising one dimer-like ‘node’ (black dashed oval) of the domain-swapped structure, as defined in the text, are shown with each in a different color. (*b*) Unit-cell contents and channels of ds-TrpR crystals. One unit cell is outlined in light blue, with the single polypeptide chain corresponding to the asymmetric unit represented in dark blue and the other polypeptide chains represented by gray ribbons. The orientations of the upper and lower assemblies correspond to those of the upper and lower schematics in (*c*). The continuous channel identified by *MOLE* analysis (*MOLEonline* 2.5; Sehnal *et al.*, 2013 ▸; Pravda *et al.*, 2018 ▸) as described in the text is represented by overlapping pale red spheres of radius 26.3–26.8 Å that fill two half-unit cells. (*c*) Schematic representation of the solvent channels in their presumed orientation with respect to the macroscopic shape of TrpR crystals. Left, side view (top) and top view (bottom) of the hexagonal bipyramidal crystals corresponding to the orientations in (*b*). Right, cartoons of the channels illustrating that they continue as linear pores through the crystal.

The present investigation aims to build on this body of work to develop a system that incorporates small to medium-sized proteins by soaking into a protein host crystal (hostal; Fig. 1 ▸). The work reported here explores the first step required for future applications, the incorporation of guest proteins into host protein crystals, and is to our knowledge the first example of the successful soaking of proteins into existing protein crystals. Crystalline tryptophan repressor protein from *Escherichia coli* (TrpR; Lawson *et al.*, 2004 ▸; Fig. 2 ▸ *a*) in an extended domain-swapped network represents a host with favorable properties, as described in this work. Horse heart cytochrome *c* (CytC; 12 kDa) and fluorophore-labeled human calmodulin (CaM; 17 kDa) are used as spectroscopically detectable guest proteins. Incorporation is monitored in time and space by conventional light microscopy and CLSM. This approach allowed the estimation of the volume fraction of the guest protein CaM in the solvent channels as a function of soaking time, indicating that very high guest contents are achievable to meet a basic requirement for structure determination. Diffraction analysis was used to compare the resolution of the TrpR crystals before and after soaking and to evaluate their applicability for guest-protein structure determination. The results show that TrpR is a useful host system for guest-protein entrainment; however, structural information about the guest proteins could not be extracted using conventional crystallo­graphic methods, and several ways are suggested in which the system could be further developed to reach this goal.

## 2. Materials and methods   

### 2.1. Preparation of TrpR (Val58Ile) as a host crystal   

A TrpR variant (Val58Ile) with properties very similar to the wild type in the domain-swapped TrpR (ds-TrpR) crystal form was used throughout this work (Sprenger *et al.*, submitted). The expression and purification of TrpR (Val58Ile) are described in Supplementary Section S1.1. Crystallization was performed using the hanging-drop vapor-diffusion technique according to previously reported conditions, with a reservoir consisting of 27.5–35%(*v*/*v*) 2-propanol, 100 m*M* NaCl, 100 m*M* HEPES pH 7.5 (Lawson *et al.*, 2004 ▸). Crystals grew within 1–2 days in drops consisting of 2–4 µl of a 1:1 mixture of TrpR (Val58Ile) solution and reservoir solution. This protein is referred to as TrpR or ds-TrpR throughout. TrpR lacks cysteine residues and is thus unreactive towards the maleimide dyes used in this work.

### 2.2. Guest-protein preparation (CaM and CytC)   

The cloning, expression and purification of Ser17Cys CaM allowing site-specific fluorophore labeling (see Section 2.2.1) was performed as described in detail previously (O’Connell *et al.*, 2010 ▸). Wild-type human CaM (UniProt ID P0DP23; molecular mass 16 828 Da) was cloned, expressed and purified as described previously (Waltersson *et al.*, 1993 ▸; O’Connell *et al.*, 2010 ▸). Cytochrome *c* from horse heart (CytC; molecular mass 12 384 Da) was purchased from Sigma–Aldrich in the form of a lyophilized powder with a purity of >99% according to the supplier’s information.

#### 2.2.1. Fluorophore labeling of Ser17Cys CaM   

Ser17Cys human CaM was originally prepared (O’Connell *et al.*, 2010 ▸) to enable the specific labeling of CaM at a site that is solvent-exposed in all known CaM target structures with fluorophores that couple to the unique cysteine using maleimide chemistry. Fluorophore labeling was performed essentially as described in O’Connell *et al.* (2010 ▸). Purified Ser17Cys CaM was dissolved in 10 m*M* phosphate, 150 m*M* NaCl, 5 m*M* DTT pH 8.0 at a protein concentration of ∼500 µ*M* and incubated for 1 h at room temperature. DTT was removed by gel filtration in 10 m*M* phosphate, 150 m*M* NaCl pH 8.0. The collected protein was incubated for 2 h at room temperature with 1 m*M* Alexa Fluor 532 Maleimide or Texas Red C_2_ Maleimide followed by gel filtration twice in water to remove free dye. The labeled protein samples were then lyophilized.

### 2.3. Protein incorporation into the hostal by soaking   

Prior to adding the guest protein for soaking, 2–4-day-old crystals with a largest dimension of 30–100 µm were first transferred into 2–3 µl drops containing only reservoir solution for 1–2 days. In the initial soaking trials, staining of the crystals was followed using a bright-field microscope. Solid CytC (as purchased in lyophilized form) or Texas Red-labeled CaM (lyophilized) were added with a spatula to the crystal drop until no more solid material dissolved during addition (see Supplementary Fig. S1).

#### 2.3.1. Incorporation of CytC for imaging   

For the imaging of CytC incorporation using CLSM, a CytC soaking solution (150 mg ml^−1^ CytC in 15.6% 2-propanol, 100 m*M* NaCl, 100 m*M* HEPES pH 7.5) was prepared by mixing a solution of 300 mg ml^−1^ CytC in 100 m*M* HEPES, 100 m*M* NaCl pH 7.5 with reservoir solution in a 1:1 ratio. 2–4 µl of this CytC soaking solution was applied as a droplet on a new cover glass, into which TrpR host crystals of 20–50 µm were transferred. The crystals in the soaking solution were placed as hanging drops over 500 µl reservoir solution to prevent evaporation. Crystals were transferred to microscope slide wells with the soaking solution (total volume of 4.5 µl) just prior to imaging.

#### 2.3.2. Incorporation of Alexa532-CaM for imaging   

For soaking studies with Alexa532-CaM, 30 µ*M* of this protein in H_2_O was mixed in a 1:1 ratio with reservoir solution, resulting in a soaking solution consisting of 15 µ*M* Alexa532-CaM, 15.6%(*v*/*v*) 2-propanol, 50 m*M* NaCl, 50 m*M* HEPES pH 7.5. To follow soaking for the initial 40 min, crystals grown in 3–4 µl hanging drops were transferred using a crystal loop into a microscope slide well containing 4.5 µl Alexa532-CaM soaking solution. To follow soaking for longer times (>1 day), the crystals were transferred into new drops containing the Alexa532-CaM soaking solution and were kept as hanging drops over 500 µl reservoir solution to prevent evaporation. The crystals were transferred to microscope slide wells with the soaking solution just prior to imaging.

For experiments with higher guest-protein concentrations and long soaking times (weeks), a protein stock solution of CaM (220 mg ml^−1^, *i.e.* 14 m*M*, CaM with 0.2% Alexa532-CaM in 10 m*M* Tris–HCl pH 7.5) was mixed in a 1:1 ratio with reservoir solution to give a final soaking solution concentration of 110 mg ml^−1^ CaM with 0.1% Alexa532-CaM in 5 m*M* Tris–HCl, 15.6% 2-propanol, 50 m*M* NaCl, 50 m*M* HEPES pH 7.5. TrpR crystals in 3–4 µl hanging drops were first transferred with a crystal loop into a new drop containing 3–4 µl of this solution and, after seven or 15 days of soaking, were subsequently transferred to a microscope slide with the soaking solution. For imaging in the initial phase of soaking, TrpR crystals were transferred from the hanging drop into a well containing 4.5 µl soaking solution consisting of 88 mg ml^−1^ (5.5 m*M*) CaM with 0.1% Alexa532-CaM and were visualized after 5 min of soaking.

### 2.4. CLSM monitoring of guest-protein incorporation   

For CLSM imaging, TrpR host crystals or protein solutions were transferred into ten-well microscope slides with 30 µm depth (Thermo Fisher) and covered with a cover slip to avoid evaporation, thus allowing imaging over longer time periods of up to 15 days. Crystals were either transferred from the drop using 0.2–0.3 mm mesh LithoLoops (Molecular Dimensions) or by pipette (0.5–1 µl) into a well pre-filled with either reservoir solution (control) or the corresponding soaking solution (see Section 2.3) to match the condition of the drop used for soaking. After transfer of the soaked TrpR crystals from the hanging drop, each microscope slide well contained 5–50 crystals or crystal debris with size range 20–60 µm. To obtain a standard curve for estimation of the CaM guest-protein concentration in the host crystal (this curve was only used for quantification for soaks with low CaM concentration), solutions of 0.3–30 µ*M* Alexa532-CaM, obtained from diluting 30 µ*M* Alexa532-CaM (in H_2_O) with reservoir solution, were applied to the wells and measured with CLSM using the same settings as for the later imaging of host crystals soaked with low concentrations of CaM. The final volume in the wells was 4.5 µl. The slides were mounted on a Leica SP5-X MP CLSM (Leica Microsystems, Heidelberg, Germany) and images with 355 nm excitation were taken using a 40× water-immersion objective (Leica; HCX APO L U-V-I 40×, 0.80 W). For imaging with 405 nm excitation an inverted Leica SP5 CLSM (Leica Microsystems, Heidelberg, Germany) was used, as this wavelength was not available using the SP5-X MP. Emission was recorded in 10 nm intervals as λ scans between 380 and 770 nm and between 415 and 755 nm, respectively.

For Texas Red soaking, imaging was carried out with excitation at 594 nm and detection of fluorescence emission between 615 and 674 nm (Supplementary Section S2.1). Crystals soaked with Alexa532-CaM or 110 mg ml^−1^ CaM with 0.1% Alexa532-CaM were imaged using an excitation wavelength of 528 nm and emission at 550–598 nm and the same CLSM settings as for all crystal imaging experiments. All images for different crystals and those used for quantification were recorded using identical settings for gain, pinhole, zoom and laser intensity as for the reference solutions of Alexa532-CaM. Images were analyzed using *ImageJ* within the *Fiji* software package and *Leica Application Suite X* (*LASX* 3.3.0).

### 2.5. X-ray data collection, processing and molecular-replacement analysis   

Diffraction data from guest-protein-soaked crystals were collected on the BioMAX beamline at MAX IV. TrpR host crystals (maximum size 50 µm) soaked for two weeks with 110 mg ml^−1^ CaM (with 0.1% Alexa532-CaM) or 150 mg ml^−1^ CytC (from the same drops as used for CLSM) were cryocooled with liquid N_2_ after additional soaking for <1 min with 25% ethylene glycol as a cryoprotectant, which was also added to the respective CaM or CytC soaking solution used for crystal soaking. In data sets from the protein-soaked crystals, 4000 frames were collected with 0.1° rotation and a detector distance set to a diffraction limit of 2.0 Å at 0.97 Å wavelength. Processing was performed with *XDS* (Kabsch, 2010 ▸) in space groups *P*1 and *P*6_1_22, using unit-cell parameters from an initial *XDS* run. Molecular replacement (MR) using *MOLREP* (Vagin & Teplyakov, 2010 ▸) was followed by refinement in *REFMAC*5 (Murshudov *et al.*, 2011 ▸) but the structures were not manually remodeled. Automated detecting of twinning and twin refinement was disabled in *REFMAC*5. Prior to the calculation of maps on an absolute scale of e^−^ Å^−3^ (END maps; Lang *et al.*, 2014 ▸), the data sets were subjected to additional refinement with *phenix.refine* (Afonine *et al.*, 2012 ▸), which was also used to generate maps with and without bulk-solvent correction (BSC). For structure determination in *P*1, an assembly of 12 monomers constituting the full unit cell of the TrpR structure (PDB entry 1mi7; Lawson *et al.*, 2004 ▸) was used as a search model. Although molecular-replacement searches were not strictly necessary for phasing because the structure of the host crystals is known, they were carried out to make sure that the structures could be refined with the same protocols regardless of any possible slight changes in packing due to presence of the guest. Molecular-replacement attempts using the guest proteins as the search model were carried out including the host as a fixed model. The channel electron density from the 2*F*_o_ − *F*_c_ and END maps was analyzed in *Coot* (Emsley *et al.*, 2010 ▸). For the purpose of visualization, map blurring was applied to the 2*F*_o_ − *F*_c_ maps with a blurring factor of 150 Å^2^ on a map displayed at a contour level of 0.5 r.m.s.d. using the map-sharpening tool in *Coot*.

## 3. Results   

### 3.1. Properties of ds-TrpR as a hostal   

Native TrpR is an extensively intertwined dimer (Schevitz *et al.*, 1985 ▸) that forms a closed structure by partial domain swapping that does not propagate beyond the dimer. In the presence of some alcohols the TrpR dimers partially unfold, associate and crystallize in the form of propagated multimeric arrays (Lawson *et al.*, 2004 ▸), in which extended polypeptide chains are connected by domain swapping throughout the crystal lattice into dimer-like ‘nodes’ (Fig. 2 ▸ *a*) in a process akin to runaway domain swapping (Guo & Eisenberg, 2006 ▸). These domain-swapped TrpR (ds-TrpR) crystals have a solvent content of ∼75% that is present in linear channels of ∼6 nm diameter along the 6_1_ screw axis of the hexagonal crystal lattice (space group *P*6_1_22; Lawson *et al.*, 2004 ▸; Figs. 2 ▸ *b* and 2 ▸ *c*). The crystals meet the chemical definition of a gel (Jones *et al.*, 2008 ▸), in that they have chain entanglement and a high solvent content, but are paradoxically ordered in a crystal lattice. Despite their very high solvent content, the ds-TrpR crystals are shown here to be mechanically stable and to tolerate changes of the environment without physical breakage or significant loss of diffraction quality, presumably due to the extensive domain swapping. Crystals of ds-TrpR are furthermore readily reproduced and grown within a day to crystal sizes ranging from microcrystals (<20 µm) to large crystals (>500 µm) depending on the 2-propanol and TrpR concentrations.

A more detailed analysis of the channel dimensions of ds-TrpR crystals was performed in the present work using *MOLEonline* 2.5 (Sehnal *et al.*, 2013 ▸; Pravda *et al.*, 2018 ▸), which takes into account side-chain hydration and flexibility (Pravda *et al.*, 2018 ▸). This program was originally designed to identify voids within a protein structure that represent, for example, potential substrate-binding pathways in enzymes or pores in membrane proteins. Here, it was used to identify the limiting radius of the ds-TrpR crystal solvent channels. Based on the analysis of an assembly covering at least one complete unit cell, ds-TrpR channels have a minimal diameter of 5.26 nm (Fig. 2 ▸ *b*). The channel size may thus be suitable to host small to medium-size guest proteins with a radius of gyration up to ∼5 nm, corresponding to a molecular mass of up to ∼50 kDa, depending on shape and dynamics (Erickson, 2009 ▸).

### 3.2. Guest-protein incorporation visualized using bright-field microscopy   

CytC (12 kDa) and CaM (17 kDa) have been extensively studied by X-ray crystallography and NMR, and their dimensions and the following specific properties suggest that they may be suitable as guests. Both proteins are highly soluble in aqueous buffers and could be dissolved in the crystallization solution used to form ds-TrpR at concentrations of ≫200 mg ml^−1^ as estimated by the addition of solid protein to crystal droplets. Analysis of the crystal structure of CytC (PDB entry 1hrc; Bushnell *et al.*, 1990 ▸) and the NMR structure of calcium-free CaM (PDB entry 1cfd; Kuboniwa *et al.*, 1995 ▸) with *HYDROPRO* (Ortega *et al.*, 2011 ▸) indicate radii of gyration (*R*_g_) of 1.28 and 2.25 nm, respectively (summarized in Supplementary Table S1), sizes that in principle allow these proteins to enter the ds-TrpR solvent channels. Both proteins have suitable absorbance and fluorescence spectra. The heme group of CytC is a natural chromophore that is covalently attached to the protein via two thioether links to Cys14 and Cys18, and the Fe atom is coordinated by the protein side chains of His18 and Met80. Heme absorbance (red color) in the visible region can be used to monitor migration in TrpR crystals by bright-field microscopy, and its fluorescence can be used in confocal imaging. A cysteine-containing variant, Ser17Cys, of the otherwise cysteine-free CaM was used for specific covalent labeling with fluorescent Texas Red Maleimide or Alexa Fluor 532 (Alexa532) Maleimide dyes. Texas Red can be detected by its absorbance (blue color) in bright-field microscopy as well as by its red fluorescence at 615–674 nm with excitation at 594 nm, and Alexa Fluor 532 by its fluorescence at 550–598 nm with excitation at 532 nm.

**Figure 3:**
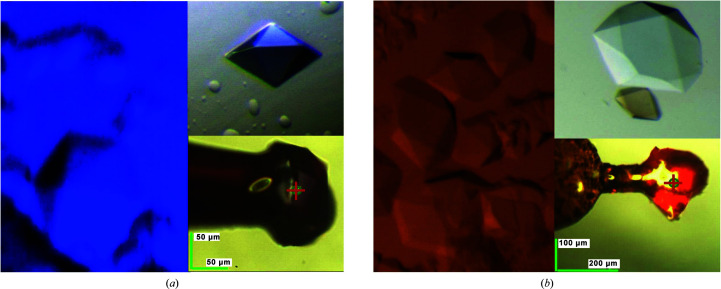
Guest-protein uptake. The left panels show bright-field microscopy images of crystal droplets taken several days after the addition of solid lyophilized protein: (*a*) Texas Red-labeled CaM, (*b*) CytC. The upper right panels show a crystal ∼1 h after transfer to a fresh reservoir droplet lacking guest protein, with a larger nonsoaked crystal included for reference in (*b*). The lower right panels show soaked crystals (50–100 µm) mounted in LithoLoops and imaged at the goniometer using the microscope camera on beamline P13, DESY, Hamburg.

The soaking procedure with CytC or CaM for bright-field microscopy as described below is shown in Supplementary Fig. S1. In brief, CytC or Texas Red-labeled CaM were added stepwise as lyophilized powders directly into droplets containing ds-TrpR crystals not older than seven days and of ∼50–300 µm in their longest dimension. Additions were continued until no more solid material went into solution within a few minutes. Staining of the ds-TrpR crystals was followed over time by bright-field microscopy, with a red or blue color detected after 1–2 days that appeared to extend throughout the crystals (Figs. 3 ▸ *a* and 3 ▸ *b*). The color of the guest proteins as judged in the bright-field microscope persists for days when the soaked crystals are transferred into new droplets without guest protein. Slight destaining is observed at the crystal edges, but the centers of the crystals remain colored for several weeks (data not shown). Crystals of ds-TrpR retained their macroscopic hexagonal bipyramidal shape throughout the staining, destaining, microscopy and diffraction analyses described below.

### 3.3. Guest-protein incorporation visualized using confocal microscopy   

CLSM was used to evaluate the distribution of the guest proteins within ds-TrpR host crystals. To evaluate the background fluorescence of the host crystals, fluorescence emission scans of nonsoaked host crystals were carried out by an SP5-X microscope at the excitation wavelength and fluorescence emission ranges of each guest protein (spectra are summarized in Supplementary Table S2). The host crystals do not show background fluorescence with 528 nm excitation. The same CLSM settings were later used for imaging Alexa532-CaM-soaked crystals. In contrast, CLSM emission scans of host crystals with a 355 or 405 nm excitation wavelength show significant fluorescence background emission between 420 and 650 nm, with a peak at ∼465 nm (Figs. 2 ▸ *a* and 2 ▸ *c*). This background fluorescence is likely to be related to ds-TrpR in crystalline form, as a solution of dimeric TrpR at 4.2 mg ml^−1^ did not show detectable emission by CLSM with the same settings and shows no absorbance above 320 nm (Supplementary Fig. S2). The origin of this autofluorescence from ds-TrpR crystals was not determined. However, lysozyme crystals also are reported to exhibit autofluorescence of unclear origin when excited in the near-UV range (Cvetkovic *et al.*, 2004 ▸), and a number of self-assembling systems have also been reported to exhibit autofluorescence (Luo *et al.*, 2001 ▸; Pinotsi *et al.*, 2013 ▸).

#### 3.3.1. Imaging of CytC in host crystals   

**Figure 4:**
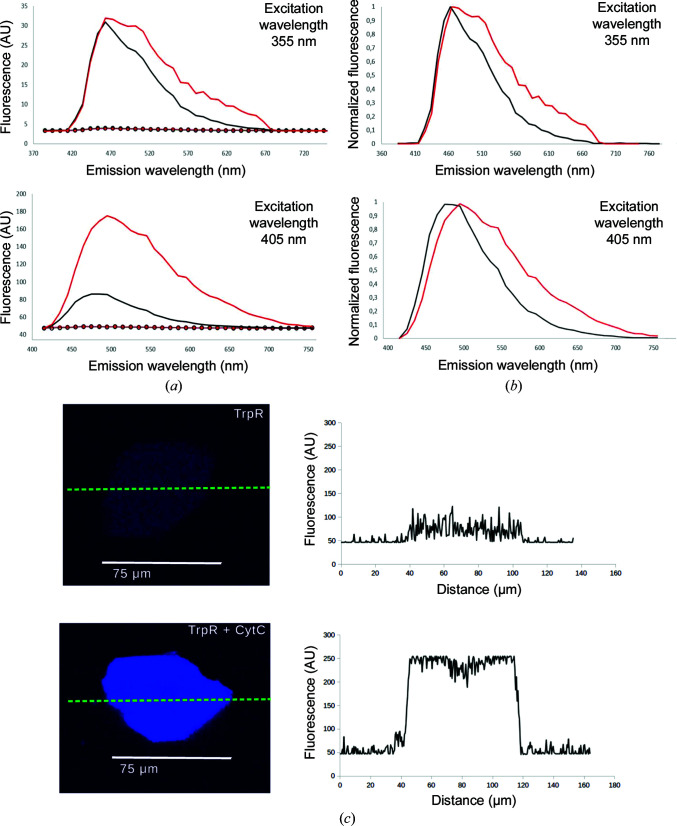
Fluorescence emission spectra of crystals with UV excitation. (*a*) Emission spectra with excitation at 355 nm with the SP5-X microscope (top) or at 405 nm with the SP5 inverted microscope (bottom). Each spectrum is the result of averaging from four or five individual crystals of nonsoaked TrpR host (black solid lines) and crystals soaked for one day with CytC (red solid lines). Dotted lines show the emission from the surrounding solution in the microscope slide well (black, 4.5 mg ml^−1^ TrpR in reservoir buffer with 15.75% 2-propanol; red, 150 mg ml^−1^ CytC in reservoir buffer with 15.75% 2-propanol). (*b*) Normalized emission spectra of crystal samples (highest emission of the sample set to one and background emission set to zero). (*c*) Upper panels, a representative nonsoaked crystal; lower panels, a representative crystal soaked for one day with 150 mg ml^−1^ CytC. Images (left) were collected with the focal plane through the center of the crystal with 405 nm excitation (SP5 inverted microsope) and emission between 614 and 790 nm. Plots of fluorescence intensities along the indicated green dotted lines are shown to the right of each crystal image.

A closer comparison of emission spectra by CLSM reveals significant differences between host crystals with and without CytC guest protein when excited in the near-UV range (Fig. 4 ▸ and Supplementary Fig. S3). With excitation at 355 nm, the emission peak is red-shifted from ∼465 to ∼475 nm and a new peak is detected at 510 nm with ∼25% increased intensity at this wavelength for CytC-soaked compared with unsoaked crystals. With excitation at 405 nm, several spectral changes are observed close to the CytC heme Soret band absorption maximum at 410 nm: the emission peak is red-shifted from ∼490 to ∼505 nm and has fourfold to fivefold higher emission intensity, a new peak is detected at 550 nm (Figs. 4 ▸ *a* and 4 ▸ *b*) and the emission intensity between ∼500 and 650 nm is increased (Fig. 4 ▸ *a*). Although CytC quantification is complicated by the background fluorescence of ds-TrpR crystals, confocal microscopy with 405 nm excitation was able to follow the penetration of CytC into the crystals semi-quantitatively over time and distance by taking advantage of the increase in fluorescence intensity above 600 nm, where host-crystal autofluorescence is nearly absent and CytC fluorescence can still be detected (Supplementary Table S2). Images with the focal plane through the center of crystals soaked with CytC for one day show more intense and homogeneous fluorescence intensity than the background fluorescence of nonsoaked host crystals, suggesting that the guest may be nearly uniformly distributed (Fig. 4 ▸ *c*). Crystals soaked for shorter times, and some large crystals soaked for up to two days, show higher fluorescence at the crystal edges than in the center (data not shown), as also described also for CaM soaking. After less than one week of CytC soaking the fluorescence was typically homogeneous in all focal planes of the crystals, suggesting that the guest distributes evenly throughout the crystals.

#### 3.3.2. Imaging of CaM in host crystals   

**Figure 5:**
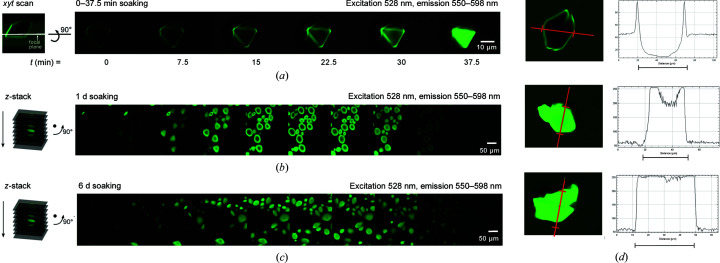
Host penetration. CLSM with excitation at 528 nm and emission at 550–598 nm was used to follow the progress of Alexa532-CaM penetration into host crystals. (*a*) Subsurface penetration. *xyt* scans taken with the focal plane set just below the surface of a single crystal, as shown in the left panel, at the indicated time points (right panel) after transfer of the crystal into reservoir solution containing 0.26 mg ml^−1^ (15 µ*M*) Alexa532-CaM. (*b*) Depth of host penetration. *z*-stack scans for a representative single crystal as shown in the schematic in the left panel. Right panels, scans at successive *z* planes with 4 µm spacing of a microscope well containing many small crystals after one day of soaking. (*c*) Progression of host penetration. As in (*b*) after six days of soaking. (*d*) Soaking with high concentrations of CaM. CLSM images (left panels) and plots (right panels) show the fluorescence intensity from three representative crystals at the cross section indicated in the left panel at 5 min, 7 days, 15 days (from top to bottom) after soaking with ∼100 mg ml^−1^ CaM including 0.1% Alexa532-CaM.

Host crystals of ∼20–∼50 µm in the longest dimension were soaked with 0.26 mg ml^−1^ (15 µ*M*) Alexa532-CaM and fluorescence was followed in the SP5-X microscope with excitation at 528 nm and emission around the maximum between ∼550 and 589 nm. Crystals soaked with the guest protein for a few minutes show Alexa532 fluorescence only in focal planes at or close to the surface (Fig. 5 ▸ *a*). In contrast, the small-molecule dye Texas Red Maleimide alone shows rapid staining throughout the entire crystal on this timescale (Supplementary Fig. S4). After one day of soaking, Alexa532-CaM fluorescence is strongest at a depth of 10–15 µm from the surface of ∼40 µm crystals. After six days, confocal images show an even distribution of the guest throughout the crystals, with increased Alexa532-CaM fluorescence at the focal plane through the center (along the *z* dimension) of the crystals compared with the images after one day of soaking (Figs. 5 ▸ *b* and 5 ▸ *c*).

Alexa532-CaM fluorescence in the crystal has the same spectrum as Alexa532-CaM in solution and was used to quantify the guest in the host crystal by assuming that the dependence of fluorescence intensity on concentration is the same in crystals as in solution. A standard curve for Alexa532-CaM in solution determined using the confocal microscope with the same settings (Supplementary Fig. S6) was linear over the observed range of intensities. From this curve, the uniform fluorescence of the crystals after six days of soaking corresponds to ∼0.12 mg ml^−1^ (7 µ*M*) Alexa532-CaM.

#### 3.3.3. Enrichment of CaM guest in host crystals   

To permit the structure determination of a guest in the channel, it is expected that higher concentrations of the guest proteins are needed than those used in the initial soaking experiments. Using only labeled CaM would bring the fluorescence intensity above the linear range of detection for the microscope under the settings used. Therefore, concentrated solutions of un­labeled CaM were supplemented with ∼0.1% Alexa532-CaM to allow the CaM distribution in the crystals to be followed by CLSM using the same settings for all images of the time series described below. The fluorescence and the corresponding plot of intensity from a focal plane through the center of a crystal imaged 5 min after transfer into 88 mg ml^−1^ CaM with 0.1% Alexa532-CaM shows that fluorescence is initially detected only at the surface of the crystal (Fig. 5 ▸ *d*). The maximum fluorescence intensity there is 95 arbitrary units (AU), compared with 45 AU for the surrounding CaM solution and zero or negligible fluorescence at the center of the crystal. After one week of soaking with 110 mg ml^−1^ CaM containing 0.1% Alexa532-CaM, a host crystal of ∼40 µm in the longest dimension shows increased fluorescence throughout (Fig. 5 ▸ *d*). The fluorescence within 5–10 µm of the crystal surface (250 AU, implying a guest concentration of >400 mg ml^−1^, *i.e.* >25 m*M*) exceeds the linear response range of the standard curve under the settings used. The center of the crystal has greatly increased fluorescence compared with the soaking solution, with a minimum fluorescence intensity of 200 AU, which is also above the linear range of the standard curve. After two weeks of soaking, the Alexa532-CaM fluorescence exceeds the linear response range throughout the entire inside of the crystal (Fig. 5 ▸ *d*). This result indicates that the host crystals become enriched in CaM relative to the surrounding CaM solution.

### 3.4. Guest-protein content in crystal channels   

**Figure 6:**
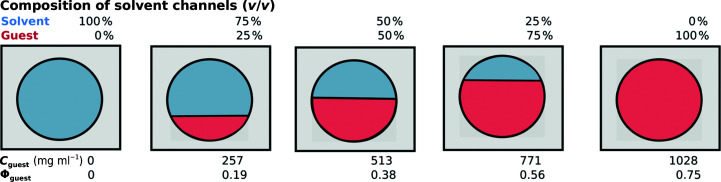
Guest content and concentration. Shown is a hypothetical unit cell (black square) of the host crystal with 25% host protein (gray, comparable to ds-TrpR crystals) and solvent channel (circle) containing solvent (blue) and guest protein (red). Matching the individual panels, the composition of the solvent channels with solvent or guest protein [%(*v*/*v*)], respectively, is given above each panel. The corresponding concentrations of guest protein in the host crystal and fractional guest content Φ_guest_ (derived from equation 2) are given below each panel. Note that for simplicity only one unit cell is shown, but the guest content can be extrapolated to a crystal (array of unit cells) when the guest concentration distribution is nearly homogeneous throughout the crystal.

For the hostal system to be developed in the future towards crystallographic structure determination of protein guests, the guest-protein content must represent a sufficient fraction of the solvent-channel volume (illustrated pictorially in Fig. 6 ▸). Guest-protein content is distinct from crystallographic occupancy, which requires the guest to not only be present but also crystallographically ordered. Guest-protein content within the host crystal can be defined analogously to the solvent content of a protein crystal. Φ_guest_ describes the fraction of the volume (*V*_guest_) of the crystal occupied by the guest protein, 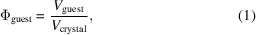
where *V*_crystal_ = *V*_host_ + *V*_solvent_ + *V*_guest_.

The total volume of the crystal, *V*_crystal_, which is fixed, is the sum of the volume of the TrpR host (itself fixed at 25% because unsoaked host crystals have 75% solvent content) plus the volumes of solvent and guest. The guest content [in %(*v*/*v*)] within the host crystal derives from Φ_guest_ {the guest content [%(*v*/*v*)] equals 100 × Φ_guest_}. The volume fraction of the guest protein in the crystal, Φ_guest_, is obtained from the experimentally estimated mass concentration of the guest protein in the crystal and the density of the guest protein (ρ_guest_):
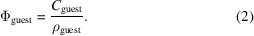
Equation (2) allows the determination of Φ_guest_, from which the guest content in the crystal can be determined assuming a homogeneous distribution of the estimated protein concentration throughout the crystal solvent. The theoretically and experimentally determined densities of proteins range from ∼1.22 to ∼1.50 g cm^−3^ depending on the molecular weight. For proteins of molecular weight <20 kDa the experimental density is generally close to the value of 1.37 g cm^−3^ (Fischer *et al.*, 2004 ▸) determined by Squire & Himmel (1979 ▸) and Gekko & Noguchi (1979 ▸). Defining guest content in the solvent channels is also useful, as the solvent channel is the only accessible space for the guest proteins and the maximal volume for the guest cannot exceed the solvent-channel volume (100% of the solvent-channel volume = 75% of the crystal volume). Thus, the volume fraction of guest in the solvent channel (Φ_guest,channel_) can be determined by dividing the estimated concentration in mg ml^−1^ measured in the crystal by the average protein density of 1.37 × 10^3^ mg ml^−1^ and correcting for the solvent fraction, 75%. Fig. 6 ▸ shows examples of guest contents in the solvent channels resulting from various guest concentrations versus the corresponding values of Φ_guest_ in the crystal solvent using equation (2).

Soaking with low concentrations of Alexa532-CaM results in 0.12 mg ml^−1^ guest protein in the host, corresponding to a guest content of only <0.01%. As described above, high Alexa532-CaM concentrations during soaking resulted in guest-protein enrichment in the channels compared with the concentration in the soaking solution, with the guest concentration estimated to be at least 400 mg ml^−1^ after two weeks of soaking (see also Supplementary Section S2.2). This concentration corresponds to a guest content in the crystals of ∼30% (a 400 mg ml^−1^ guest concentration corresponds to a volume fraction of guest Φ_guest_ = 0.29 and Φ_guest,channel_ = 0.39). Although there are many possible sources of error in this estimate, its order of magnitude is similar to the TrpR content in the crystals of 25% (volume fraction 0.25), suggesting that long soaking times with high concentrations of the guest achieve guest contents that may support crystallographic structure determination if the guests are sufficiently ordered in the crystal.

Furthermore, crystal staining with CytC followed by bright-field microscopy, and its fluorescence spectral shift in CLSM indicating uptake, suggest that CytC also enters host crystals, although following the CytC guest-protein distribution quantitatively over time by CLSM and estimation of its content were challenging. The slightly smaller radius of gyration and the larger diffusion coefficient of CytC suggest that its migration in the crystals should be at least as facile as that of CaM, although the unknown factors leading to enrichment of CaM in the crystals may differ for CytC.

### 3.5. X-ray diffraction of soaked crystals   

Several crystals of ∼20–50 µm in the longest dimension were harvested after two weeks of soaking with either 110 mg ml^−1^ CaM (with an estimated guest concentration of 400 mg ml^−1^ in the channels as described above) or 130 mg ml^−1^ CytC. In each case crystals were from the same drops as used for CLSM. Crystals were tested by X-ray diffraction on the BioMAX beamline at MAX IV, Lund. Reference diffraction data for nonsoaked host crystals alone, set up for growth at the same time as the crystals for soaking, were measured during the same experiment. All data sets were processed in *P*6_1_22 and also in *P*1 (Supplementary Table S6 shows average and standard deviations of unit-cell parameters and *R*_meas_ for data sets from soaked and unsoaked crystals; full parameters and processing statistics are given in Supplementary Tables S3–S5 and S7–S9). The choice to additionally carry out structure determination in *P*1 was made in order to consider the additional possibility that the guests may follow the same lattice but not the same space-group symmetry as the host. Data sets were collected for four individual CaM- and CytC-soaked crystals with maximal resolutions of 2.45–2.90 Å and 2.75–2.95 Å, respectively (cutoff according to the shell with CC_1/2_ > 0.5 in the first *XDS* run in either *P*6_1_22 or *P*1). The resolution of the soaked crystals is only slightly decreased (maximal resolution 2.45 Å) compared with the test set of four nonsoaked ds-TrpR host crystals measured in the same experiment (2.2 Å maximal resolution). The unit-cell parameters show no large differences among the data sets, but *R*_meas_ for merging in *P*6_1_22 is higher, especially for the CytC-soaked crystals (>15%) compared with nonsoaked ds-TrpR crystals (∼12%) (Supplementary Table S6). These differences in the merging statistics may directly or indirectly be a result of the soaking procedure and may be caused by, for example, crystal deterioration or contributions to the diffraction by a guest that may not follow the host symmetry perfectly. However, similarly as for merging in *P*6_1_22, merging in *P*1 also results in an elevated *R*_meas_ for the soaked crystals (∼13% versus ∼10% for nonsoaked crystals; Supplementary Table S6). Analysis by *phenix.xtriage* in the *Phenix* suite (Liebschner *et al.*, 2019 ▸) and *POINTLESS* (Evans, 2011 ▸) excluded twinning as a possible explanation for the high *R*_meas_ of the soaked compared with unsoaked crystals, and showed similar correlation coefficients for reflections related by the different rotational symmetry operators, suggesting that the guest is not in a different lattice to the host.

The structures of ds-TrpR from the best-diffracting crystals from each soaking experiment and from nonsoaked crystals were determined by molecular replacement in *P*6_1_22 as well as in *P*1 using a previously determined ds-TrpR crystal structure refined at 2.05 Å resolution (PDB entry 6st6) as a search model. The host structure solved in *P*1 does not show any obvious differences compared with the *P*6_1_22 structure, showing that a breakdown in symmetry is unlikely to be the origin of the higher merging statistics described above. Refinement statistics are summarized in Table 1 ▸ (complete refinement statistics are given in Supplementary Tables S7–S9). The *R*_free_/*R*_work_ values are slightly elevated for the guest-soaked compared with unsoaked ds-TrpR crystals. The elevated values may reflect several factors, including differences in resolution or some contribution from the guest. However, the effect is over the full resolution range and is not the result of badly modeled low-resolution data (Supplementary Table S7), and thus does not seem to be caused by erroneous bulk-solvent correction in the guest-soaked crystals.

**Figure 7:**
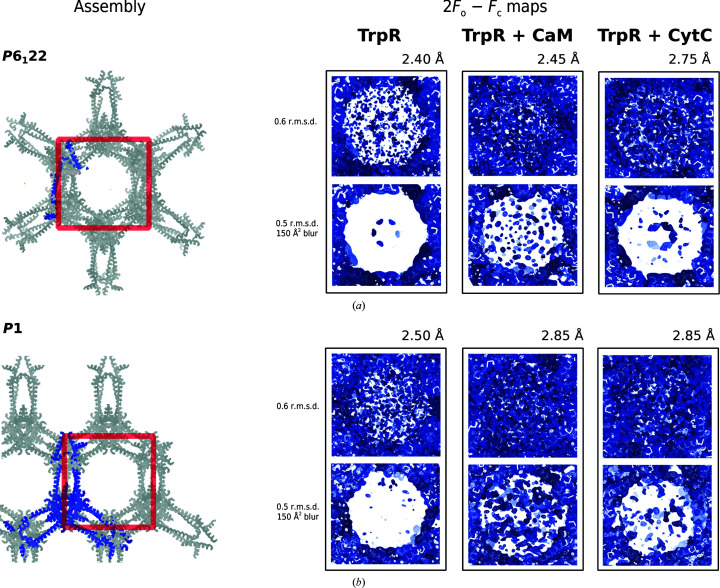
Guest electron density in solvent channels. Diffraction data were obtained from crystals like those in Fig. 5 ▸(*d*) after 15 days of soaking with Alex-CaM or CytC at BioMAX, Lund and were compared with nonsoaked crystals measured in the same experiment. The data sets were processed in *P*6_1_22 (*a*) or *P*1 (*b*). Left, protein-assembly cartoons (gray ribbons) were generated from the molecule(s) (blue ribbon) in the asymmetric unit surrounding each channel area marked with a red square. Right, electron density within the channel areas corresponding to each red square on the left for ds-TrpR crystals alone or soaked with Alexa532-CaM or CytC as indicated. Each structure was solved at the indicated resolution. Electron density is represented as 2*F*_o_ − *F*_c_ maps at a 0.6 r.m.s.d. contour level (upper panels) or at a 0.5 r.m.s.d. contour level with blurring to 150 Å^2^ (lower panels, blurring as explained in Section 2). The images were generated using *Coot*.

Molecular-replacement searches readily identified the ds-TrpR host in crystals soaked with CaM or CytC, but no solution could be found for the guests when their structures were included as search models in molecular replacement (Section S2.3). Weak electron density detected in the solvent channels could not be identified as partial or complete structures of CaM or CytC in the corresponding soaked crystals. To visualize this weak density, the electron density is shown in Fig. 7 ▸ as 2*F*_o_ − *F*_c_ maps at high noise levels (0.6 r.m.s.d.) and as blurred maps to enhance low-resolution information. Blurring of the 2*F*_o_ − *F*_c_ maps was used in contrast to map sharpening, which is typically performed to increase the quality of high-resolution information in crystal structures (Liu & Xiong, 2014 ▸). A blurring *B* value of 150 Å^2^ was chosen because at this value the electron density in the channels of unsoaked host crystals largely disappears but the density of the host remains well defined. For the soaked host crystals (particularly CaM), but not for the nonsoaked ds-TrpR host, features of connected electron density are visible when map blurring is applied. The observation of additional density is consistent with what is seen in comparison of maps on the absolute scale of electrons per unit volume (e^−^ Å^−3^; END maps, as described by Lang *et al.*, 2014 ▸) for better comparability between data sets (Supplementary Fig. S6). Inspection of maps after refinement without bulk-solvent correction indicates that the channel density for guest-soaked crystals is enhanced compared with nonsoaked crystals (Supplementary Fig. S6), suggesting that bulk-solvent correction may contribute to suppressing the guest density. The additional electron density observed in CaM-soaked crystals cannot be explained by the different resolutions of the data sets, as the same map features are seen when all of the data are truncated to the same (lower) resolution of 4 Å (Supplementary Fig. S8). Although these results suggest that the channel density is due to a scattering contribution from the guest protein, it is not clear at present how useful it may be for future structural interpretation.

## 4. Discussion   

### 4.1. Following incorporation of guest proteins using CLSM   

The staining of ds-TrpR crystals by the two small (<20 kDa) colored guest proteins observed by bright-field microscopy was a first indication that proteins of this size can enter the crystals, but did not allow conclusions about their penetration depth or uniformity of distribution. As indicated by the work of Cvetkovic *et al.* (2004 ▸), CLSM is a useful method to follow the diffusion of fluorescent small molecules within crystals. In the present study, CLSM imaging of the distribution of fluorescently labeled CaM in ds-TrpR crystals confirms the slow migration of the guest protein over a period of weeks to reach a uniform distribution throughout 20–50 µm host crystals. The results thus indicate that CLSM can be extended from small molecules to follow guest-protein incorporation into solvent channels of protein crystals, although the guest must be labeled if not naturally fluorescent. A limitation of this technique is the possible background fluorescence of the host crystal itself, as has been reported for lysozyme crystals (Cvetkovic *et al.*, 2004 ▸) and observed in this study for the ds-TrpR host crystals with excitation in the near-UV range. This background fluorescence hampered the quantification of CytC in ds-TrpR crystals, although the distinct emission spectrum of entrained CytC signaled incorporation and was used to follow the guest-protein distribution in the crystals semi-quantitatively. To our knowledge, the current work provides the first evidence that proteins can be incorporated via soaking into solvent channels of existing protein crystals. Using high concentrations of CaM (∼100 mg ml^−1^) for soaking results in an apparently at least fourfold higher guest-protein concentration in the crystal compared with the soaking solution, suggesting an enrichment effect of the guest in the mesoporous crystals of the host. Enrichment of proteins and peptides in silica-based mesoporous materials has previously been reported (Qian *et al.*, 2013 ▸).

It will be useful to better understand and develop models for the migration of proteins through a protein-based mesoporous system such as the ds-TrpR crystals used here. Unconstrained diffusion times for single protein molecules based on Brownian motion (Smoluchowski, 1906 ▸; Nelson, 1967 ▸) suggest a timescale of seconds for proteins to move a distance of 50 µm, the maximal dimension of most of the crystals used in these experiments. This rough estimate is for free diffusion, which is not the case here, and it neglects many factors, including the concentration gradient between the bulk solution and the crystal solvent channels, the channel architecture and composition, solvent viscosity in the channels and molecular interactions between the host and guest. Diffusion in bulk solvent differs from migration of proteins in channels of dimensions similar to the proteins themselves because proteins limit the motions of others in the same channel. Experimental studies of small-molecule diffusion through protein crystals showed that in several cases equilibrium is reached only after several hours (Geremia *et al.*, 2006 ▸) and that ligand occupancy in binding sites continues to increase over hours of soaking for some crystals (Collins *et al.*, 2017 ▸). It has further been observed that the soaking time needed to reach full ligand occupancy depends on the crystal size and on the organization and size of the solvent channels (Velev *et al.*, 2000 ▸; Mizutani *et al.*, 2014 ▸). Thus, modeling the apparent diffusion process is likely to be very challenging even for smaller molecules, emphasizing the importance of experimental benchmarking on a case-by-case basis. Given the long apparent diffusion times observed for some small molecules, the even longer times for CaM observed here are not surprising. Future experiments that explore the apparent diffusion coefficients of guest proteins in the channels, for example by using fluorescence recovery after photobleaching, may eventually allow the modeling of guest migration based on experimental data, as has been performed for a few small-molecule cases (Velev *et al.*, 2000 ▸; Cvetkovic *et al.*, 2004 ▸; Geremia *et al.*, 2006 ▸; Malek, 2007 ▸).

### 4.2. ds-TrpR crystals as a hostal system   

The CLSM studies suggest that guest proteins become distributed uniformly within the ds-TrpR host crystals and enriched within the channels after soaking for two weeks at high guest concentrations. These treatments demand crystal robustness, as they involve considerable changes of the crystal environment. Guest-soaked ds-TrpR host crystals diffract to resolutions similar to nonsoaked host crystals (the highest resolution for unsoaked host crystals used in this study is about 2.2 Å, versus 2.45 Å for guest-soaked host crystals), and ds-TrpR crystals do not develop crystal pathologies such as twinning upon soaking. Despite the very high solvent content of ds-TrpR crystals, they are shown here to be mechanically stable and to tolerate these changes in the environment without physical breakage or significant loss of diffraction quality. These observations from the soaking studies show that the crystals can tolerate at least some chemical changes in the surrounding solution. Future work may therefore explore whether host–guest interactions can be promoted by modification of the crystal chemical environment, for example, by manipulation of the Hofmeister series of ions that may favor or disfavor electrostatic or hydrophobic interactions.

### 4.3. Guest requirements   

Guest proteins of up to ∼36 kDa could theoretically follow the hexagonal symmetry of ds-TrpR crystals, as estimated very roughly (a TrpR monomer of 12 kDa comprising 25% crystal content with 75% solvent content implies 12 kDa TrpR plus 36 kDa guest comprising 100% crystal content with ∼0% solvent). This is an extreme upper limit, however, considering that 0% solvent content is not realistic and that the pore shape also contributes to the space available for the guest. Thus, guest proteins significantly smaller than 35 kDa are most likely to meet the requirements of size and symmetry for the ds-TrpR hostal system. As the guest proteins studied here, CytC (12 kDa) and CaM (17 kDa), are well below this limit, they are theoretically suited to penetrate the channels and follow the 6_1_22 symmetry of ds-TrpR with a rough estimate of ≥37% solvent content at maximal guest content, although they need not fully or partially follow the 6_1_22 symmetry of the ds-TrpR host. Guests of up to ∼50 kDa could in principle be accommodated in the same primitive lattice as the host but without internal symmetry (*P*1). The larger the guest, the higher the likelihood that the guest will not follow the host symmetry because there are fewer possible ways to occupy the limited space, which also depends on the guest shape. In the diffraction data sets analyzed here there was no indication that the guests are ordered but do not follow the host symmetry or occupy a lattice different from that of the host.

### 4.4. Prospects for structure determination   

The hostal system reported here is conceptually related to the crystal sponge host–guest systems described by Inokuma *et al.* (2013 ▸), as it relies on the incorporation of guest molecules by soaking into a crystalline host. Sponge systems exploit the small channels of crystalline hosts to accommodate, and optimally to order, small guests, although the fundamental principles of host–guest interactions for the sponge method are not fully understood (Hayes *et al.*, 2016 ▸). Recent developments with crystal sponge hosts have allowed the structure determination of a wider range of guests including increasingly larger, more flexible and chemically complex molecules (Yoshioka *et al.*, 2015 ▸; Ning *et al.*, 2016 ▸; Rosenberger *et al.*, 2020 ▸) as well as compounds in aqueous solution (Poel *et al.*, 2019 ▸). Commonly used host–guest sponge systems are typically not designed for interactions with a specific guest, and a single crystal host may be used for guests with varying host–guest interactions (Hoshino *et al.*, 2016 ▸). Translation of the principles applying to small-molecule crystal sponges to hostal systems cannot be predicted with confidence at this time due to the larger size and thus the higher structural complexity and potentially higher flexibility of proteins compared with the typical targets of the crystal sponge method to date. Whether the crystal sponge method can be extended to hostals remains an open question that can only be answered by further experimental work, although the current study shows that the required first step, the incorporation of guest proteins into a hostal at high concentrations, is effective.

The present work shows that starting from bulk concentrations of ∼100 mg ml^−1^ CaM, concentrations of ∼400 mg ml^−1^ CaM within the crystal channels were achievable, corresponding to 29% of the crystal volume (*i.e.* 39% of the crystal solvent volume, which is 75% in the ds-TrpR crystal, is replaced by CaM) and a nearly equal molar ratio of CaM guest to TrpR host (1.1:1). Such enrichment over the surrounding bulk may be due to favorable interactions between CaM and the host channels or due to a net gain in entropy if calmodulin replaces a significant number of ordered water molecules from the channels. High guest content within the host crystals is a necessary, but not sufficient, requirement to achieve the high site occupancies needed for conventional structure determination of the guests. If all guest molecules follow the host crystallographic symmetry and have limited intrinsic flexibility or internal disorder, the concentrations achieved here with CaM are expected to yield occupancies that are sufficient for structure determination. However, structural information on the guests was not easily obtained by conventional structure-determination methods. This observation suggests that the guest has a large degree of disorder in the host channels, making the occupancy at individual crystallographic sites too low to solve the structure in conventional ways; positional disorder and/or internal guest dynamics may be responsible.

Thus, the eventual success of a hostal method for structure determination is likely to depend on improving the order of the guest molecules experimentally so as to limit the number of guest conformations and improve the occupancy at crystallographic sites. The ds-TrpR host offers several approaches to increase the order of guest proteins by promoting specific and strong host–guest interactions while allowing adequate migration of the guest through the solvent channels. One potential way to order guests involves chemical tagging with mimics of the physiological ligand l-tryptophan (l-Trp). l-Trp binds to ds-TrpR in a position equivalent to its binding site in dimeric TrpR (PDB entry 1jhg; Lawson, 1996 ▸; Sprenger *et al.*, submitted), a result that is not necessarily expected because the residues of the binding site arise from three polypeptide chains in ds-TrpR rather than two in native dimeric TrpR. This approach might be especially useful with smaller guests (for example peptides) that may be able to diffuse past the first tightly bound molecules that limit the space in the channels.

An additional feature of ds-TrpR crystals that may facilitate the ordering of guest proteins is that the N-terminus of the protein faces the crystal solvent channels (Lawson *et al.*, 2004 ▸) and might be engineered in various ways. Recent experiments yet to be reported (Sprenger *et al.*, in preparation) also reveal that TrpR bearing an N-terminal extension crystallizes in a domain-swapped form without degradation of diffraction quality and that the extension points into the solvent channels. This result suggests that engineering the host to include an N-terminal peptide representing a binding site as ‘bait’ for a peptide-binding guest protein such as CaM might enable targeting to the channels, enhancing crystallographic site occupancy that follows the symmetry of the host. In this context, the physiological interactions of CaM might be exploited as a proof of concept, as the binding of CaM or its individual domains to peptide targets is additionally dependent on calcium ions that can be used to trigger binding (Babu *et al.*, 1985 ▸; Hoeflich & Ikura, 2002 ▸; Tidow & Nissen, 2013 ▸). Triggering of guest binding after soaking aims at limiting the potential blocking of the solvent pores, thus allowing facile guest migration prior to high-affinity binding. Finally, if one anchor point is not sufficient to order a guest for crystallo­graphic analysis, subsequent chemical cross-linking of guest and host can be envisaged in addition due to the robustness of the ds-TrpR crystals to chemical conditions.

Recent advances in the fields of X-ray diffraction and the interpretation of low-level electron densities may additionally help to solve guest-protein structures in the future. The lowest limit of site occupancy for a molecule to be structurally elucidated using conventional crystallography is not completely clear, but it has been suggested (Deller & Rupp, 2015 ▸) that atoms with occupancies of 0.15 or less cannot be interpreted. Another study shows that ligands with occupancies as low as 0.22 can be modeled and refined using, for example, *PanDDA* maps (Pearce, Bradley *et al.*, 2017 ▸; Pearce, Krojer *et al.*, 2017 ▸). Guest-site occupancies of at least 0.2 with moderate *B* factors may be achievable in the future using the anchoring strategies suggested above, thus potentially making structure determination possible with careful density analysis. Diffraction methods that make use of crystal disorder may also be advantageous. It has, for example, been shown that taking the diffuse scattering signal from a diffraction experiment into account improves structure interpretation for less ordered parts of crystal structures (Chapman *et al.*, 2017 ▸).

### 4.5. Potential of hostal systems for biotechnological applications   

In addition to using ds-TrpR as a hostal system for solving the crystal structures of guest proteins, the robust crystals of ds-TrpR, and similar hostals that may be developed in the future, may also be applicable in other fields of biotechnology. Protein crystals, especially of enzymes, have gained interest, for example, for use as ‘micro-bioreactors’ that serve to concentrate reactants in confined volumes, in line with other protein-encapsulation methods (Minten *et al.*, 2009 ▸). Crystals used as bioreactors are typically not of diffraction quality, as they are stabilized to withstand harsh reaction conditions (Yan *et al.*, 2015 ▸; Hartje & Snow, 2018 ▸), for example by glutaraldehyde cross-linking (Andersen *et al.*, 2009 ▸). Protein crystals or their solvent channels have also been investigated as templates for nanomaterials or as drug-delivery systems (reviewed by Abe & Ueno, 2015 ▸). Taking advantage of the fact that the ds-TrpR hostal is quite stable even without cross-linking, this host–guest system could be useful to explore protein-encapsulation applications that do not require sufficient guest order for diffraction studies. Recently, a number of porous or protein-based cargo systems for controlled drug release have been discussed, such as mesoporous silica (Vallet-Regí *et al.*, 2017 ▸), metal–organic frameworks (Ni *et al.*, 2019 ▸) and protein cages such as ferritin (Chakraborti *et al.*, 2019 ▸). Mesoporous protein systems such as the ds-TrpR host system described here may also be useful to explore as potential cargo (guest) carriers for such applications.

## Supplementary Material

Supplementary materials and methods, supplementary results, Supplementary Tables and Supplementary Figures. DOI: 10.1107/S2059798321001078/rr5201sup1.pdf

## Figures and Tables

**Table 1:** Structure-determination and merging statistics (short version) of selected data sets in space groups *P*1 and *P*6_1_22

Crystal (ds-TrpR)	Data-set ID	*P*1				*P*6_1_22			
		Highest resolution (Å)	*R*_meas_ † (%)	*R*_work_ (%)	*R*_free_ (%)	Highest resolution (Å)	*R*_meas_ † (%)	*R*_work_ (%)	*R*_free_ (%)
Nonsoaked	Hostal_2	2.50	8.0 (151.8)‡	26.9	31.0	2.40	9.3 (248.6)	26.2	29.9
CaM-soaked	CaM_3	2.85	13.8 (122.2)	25.6	30.8	2.45	18.6 (357.1)	28.4	32.9
CytC-soaked	Cyt_2	2.85	14.7 (225.4)	26.2	30.2	2.75	16.4 (312.1)	31.2	37.6

† *R*_meas_ is the redundancy-independent merging *R* factor (*R*_merge_).

‡ Values in parentheses are for the highest resolution shell. Full data-collection, processing and structure-refinement statistics are provided in Supplementary Tables S7–S9.

## References

[bb1] Abe, S. & Ueno, T. (2015). *RSC Adv.* **5**, 21366–21375.

[bb4] Afonine, P. V., Grosse-Kunstleve, R. W., Echols, N., Headd, J. J., Moriarty, N. W., Mustyakimov, M., Terwilliger, T. C., Urzhumtsev, A., Zwart, P. H. & Adams, P. D. (2012). *Acta Cryst.* D**68**, 352–367. 10.1107/S0907444912001308 PMC3322595 22505256

[bb5] Andersen, O. A., Schönfeld, D. L., Toogood-Johnson, I., Felicetti, B., Albrecht, C., Fryatt, T., Whittaker, M., Hallett, D. & Barker, J. (2009). *Acta Cryst.* D**65**, 872–874. 10.1107/S0907444909017855 19622871

[bb6] Azuma, Y., Zschoche, R., Tinzl, M. & Hilvert, D. (2016). *Angew. Chem. Int. Ed.* **55**, 1531–1534. 10.1002/anie.201508414 26695342

[bb7] Babu, Y. S., Sack, J. S., Greenhough, T. J., Bugg, C. E., Means, A. R. & Cook, W. J. (1985). *Nature*, **315**, 37–40. 10.1038/315037a0 3990807

[bb8] Benvenuti, M. & Mangani, S. (2007). *Nat. Protoc.* **2**, 1633–1651. 10.1038/nprot.2007.198 17641629

[bb9] Blundell, T. L. (2017). *IUCrJ*, **4**, 308–321. 10.1107/S2052252517009241 PMC5571795 28875019

[bb98] Bushnell, G. W., Louie, G. V. & Brayer, G. D. (1990). *J. Mol. Biol.* **214**, 585–595. 10.1016/0022-2836(90)90200-6 2166170

[bb10] Chakraborti, S., Korpi, A., Kumar, M., Stępień, P., Kostiainen, M. A. & Heddle, J. G. (2019). *Nano Lett.* **19**, 3918–3924. 10.1021/acs.nanolett.9b01148 31117758

[bb11] Chapman, H. N., Yefanov, O. M., Ayyer, K., White, T. A., Barty, A., Morgan, A., Mariani, V., Oberthuer, D. & Pande, K. (2017). *J. Appl. Cryst.* **50**, 1084–1103. 10.1107/S160057671700749X PMC5541353 28808434

[bb12] Cole, J. C., Giangreco, I. & Groom, C. R. (2017). *Acta Cryst.* D**73**, 234–239. 10.1107/S2059798316014352 PMC5349435 28291758

[bb13] Collins, P. M., Ng, J. T., Talon, R., Nekrosiute, K., Krojer, T., Douangamath, A., Brandao-Neto, J., Wright, N., Pearce, N. M. & von Delft, F. (2017). *Acta Cryst.* D**73**, 246–255. 10.1107/S205979831700331X PMC5349437 28291760

[bb14] Cvetkovic, A., Picioreanu, C., Straathof, A. J. J., Krishna, R. & van der Wielen, L. A. M. (2005). *J. Am. Chem. Soc.* **127**, 875–879. 10.1021/ja0440708 15656625

[bb15] Cvetkovic, A., Straathof, A. J. J., Hanlon, D. N., van der Zwaag, S., Krishna, R. & van der Wielen, L. A. M. (2004). *Biotechnol. Bioeng.* **86**, 389–398. 10.1002/bit.20067 15112291

[bb16] Deller, M. C. & Rupp, B. (2015). *J. Comput. Aided Mol. Des.* **29**, 817–836. 10.1007/s10822-015-9833-8 PMC4531100 25665575

[bb17] Emsley, P., Lohkamp, B., Scott, W. G. & Cowtan, K. (2010). *Acta Cryst.* D**66**, 486–501. 10.1107/S0907444910007493 PMC2852313 20383002

[bb18] Erickson, H. P. (2009). *Biol. Proced. Online*, **11**, 32–51. 10.1007/s12575-009-9008-x PMC3055910 19495910

[bb19] Evans, P. R. (2011). *Acta Cryst.* D**67**, 282–292. 10.1107/S090744491003982X PMC3069743 21460446

[bb20] Falkner, J. C., Al-Somali, A. M., Jamison, J. A., Zhang, J., Adrianse, S. L., Simpson, R. L., Calabretta, M. K., Radding, W., Phillips, G. N. & Colvin, V. L. (2005). *Chem. Mater.* **17**, 2679–2686.

[bb21] Fischer, H., Polikarpov, I. & Craievich, A. F. (2004). *Protein Sci.* **13**, 2825–2828. 10.1110/ps.04688204 PMC2286542 15388866

[bb22] Fujita, D., Suzuki, K., Sato, S., Yagi-Utsumi, M., Yamaguchi, Y., Mizuno, N., Kumasaka, T., Takata, M., Noda, M., Uchiyama, S., Kato, K. & Fujita, M. (2012). *Nat. Commun.* **3**, 1093. 10.1038/ncomms2093 23033069

[bb23] Gekko, K. & Noguchi, H. (1979). *J. Phys. Chem.* **83**, 2706–2714.

[bb24] Geremia, S., Campagnolo, M., Demitri, N. & Johnson, L. N. (2006). *Structure*, **14**, 393–400. 10.1016/j.str.2005.12.007 16531224

[bb25] Giegé, R. (2017). *IUCrJ*, **4**, 340–349. 10.1107/S2052252517006595 PMC5571797 28875021

[bb26] Guo, Z. & Eisenberg, D. (2006). *Proc. Natl Acad. Sci. USA*, **103**, 8042–8047. 10.1073/pnas.0602607103 PMC1472426 16698921

[bb27] Hartje, L. F. & Snow, C. D. (2018). *Wiley Interdiscip. Rev. Nanomed. Nanobiotechnol.* **11**, e1547. 10.1002/wnan.1547 30488657

[bb88] Hayes, L. M., Knapp, C. E., Nathoo, K. Y., Press, N. J., Tocher, D. A. & Carmalt, C. J. (2016). *Cryst. Growth Des.* **16**, 3465–3472.

[bb28] Hoeflich, K. P. & Ikura, M. (2002). *Cell*, **108**, 739–742. 10.1016/s0092-8674(02)00682-7 11955428

[bb29] Hoshino, M., Khutia, A., Xing, H., Inokuma, Y. & Fujita, M. (2016). *IUCrJ*, **3**, 139–151. 10.1107/S2052252515024379 PMC4775162 27006777

[bb30] Inokuma, Y., Yoshioka, S., Ariyoshi, J., Arai, T., Hitora, Y., Takada, K., Matsunaga, S., Rissanen, K. & Fujita, M. (2013). *Nature*, **495**, 461–466. 10.1038/nature11990 23538828

[bb31] Jones, R. G., Kahovec, J., Stepto, R., Wilks, E. S., Hess, M., Kitayama, T. & Metanomski, W. V. (2008). *Compendium of Polymer Terminology and Nomenclature: IUPAC Recommendations 2008*. Cambridge: Royal Society of Chemistry.

[bb32] Kabsch, W. (2010). *Acta Cryst.* D**66**, 125–132. 10.1107/S0907444909047337 PMC2815665 20124692

[bb99] Kuboniwa, H., Tjandra, N., Grzesiek, S., Ren, H., Klee, C. B. & Bax, A. (1995). *Nat. Struct. Mol. Biol.* **2**, 768–776. 10.1038/nsb0995-768 7552748

[bb33] Lang, P. T., Holton, J. M., Fraser, J. S. & Alber, T. (2014). *Proc. Natl Acad. Sci. USA*, **111**, 237–242.

[bb34] Lawson, C. L. (1996). *Nat. Struct. Mol. Biol.* **3**, 986–987. 10.1038/nsb1296-986 8946848

[bb35] Lawson, C. L., Benoff, B., Berger, T., Berman, H. M. & Carey, J. (2004). *Structure*, **12**, 1099–1108. 10.1016/j.str.2004.03.019 PMC3228604 15274929

[bb3] Liebschner, D., Afonine, P. V., Baker, M. L., Bunkóczi, G., Chen, V. B., Croll, T. I., Hintze, B., Hung, L.-W., Jain, S., McCoy, A. J., Moriarty, N. W., Oeffner, R. D., Poon, B. K., Prisant, M. G., Read, R. J., Richardson, J. S., Richardson, D. C., Sammito, M. D., Sobolev, O. V., Stockwell, D. H., Terwilliger, T. C., Urzhumtsev, A. G., Videau, L. L., Williams, C. J. & Adams, P. D. (2019). *Acta Cryst.* D**75**, 861–877. 10.1107/S2059798319011471 PMC6778852 31588918

[bb36] Liu, C. & Xiong, Y. (2014). *J. Mol. Biol.* **426**, 980–993. 10.1016/j.jmb.2013.11.014 24269527

[bb37] Luo, J., Xie, Z., Lam, J. W. Y., Cheng, L., Tang, B. Z., Chen, H., Qiu, C., Kwok, H. S., Zhan, X., Liu, Y. & Zhu, D. (2001). *Chem. Commun.*, pp. 1740–1741. 10.1039/b105159h 12240292

[bb38] Malek, K. (2007). *Biotechnol. Lett.* **29**, 1865–1873. 10.1007/s10529-007-9466-7 PMC2045120 17641823

[bb39] Matthews, B. W. (1968). *J. Mol. Biol.* **33**, 491–497. 10.1016/0022-2836(68)90205-2 5700707

[bb40] McNae, I. W., Kan, D., Kontopidis, G., Patterson, A., Taylor, P., Worrall, L. & Walkinshaw, M. D. (2005). *Crystallogr. Rev.* **11**, 61–71.

[bb41] Minten, I. J., Hendriks, L. J. A., Nolte, R. J. M. & Cornelissen, J. J. L. M. (2009). *J. Am. Chem. Soc.* **131**, 17771–17773. 10.1021/ja907843s 19995072

[bb42] Mizutani, R., Shimizu, Y., Saiga, R., Ueno, G., Nakamura, Y., Takeuchi, A., Uesugi, K. & Suzuki, Y. (2014). *Sci. Rep.* **4**, 5731. 10.1038/srep05731 PMC5375977 25043871

[bb43] Murray, C. W. & Rees, D. C. (2009). *Nat. Chem.* **1**, 187–192. 10.1038/nchem.217 21378847

[bb44] Murshudov, G. N., Skubák, P., Lebedev, A. A., Pannu, N. S., Steiner, R. A., Nicholls, R. A., Winn, M. D., Long, F. & Vagin, A. A. (2011). *Acta Cryst.* D**67**, 355–367. 10.1107/S0907444911001314 PMC3069751 21460454

[bb45] Nelson, E. (1967). *Dynamical Theories of Brownian Motion*. Princeton University Press.

[bb46] Ni, K., Aung, T., Li, S., Fatuzzo, N., Liang, X. & Lin, W. (2019). *Chem*, **5**, 1892–1913. 10.1016/j.chempr.2019.05.013 PMC6681452 31384694

[bb47] Ning, G.-H., Matsumura, K., Inokuma, Y. & Fujita, M. (2016). *Chem. Commun.* **52**, 7013–7015. 10.1039/c6cc03026b 27157794

[bb48] O’Connell, D. J., Bauer, M. C., O’Brien, J., Johnson, W. M., Divizio, C. A., O’Kane, S. L., Berggård, T., Merino, A., Åkerfeldt, K. S., Linse, S. & Cahill, D. J. (2010). *Mol. Cell. Proteomics*, **9**, 1118–1132. 10.1074/mcp.M900324-MCP200 PMC2877974 20068228

[bb49] Ortega, A., Amorós, D. & García de la Torre, J. (2011). *Biophys. J.* **101**, 892–898. 10.1016/j.bpj.2011.06.046 PMC3175065 21843480

[bb50] Pearce, N. M., Bradley, A. R., Krojer, T., Marsden, B. D., Deane, C. M. & von Delft, F. (2017). *Struct. Dyn.* **4**, 032104. 10.1063/1.4974176 PMC5336473 28345007

[bb51] Pearce, N. M., Krojer, T., Bradley, A. R., Collins, P., Nowak, R. P., Talon, R., Marsden, B. D., Kelm, S., Shi, J., Deane, C. M. & von Delft, F. (2017). *Nat. Commun.* **8**, 15123. 10.1038/ncomms15123 PMC5413968 28436492

[bb52] Pinotsi, D., Buell, A. K., Dobson, C. M., Kaminski Schierle, G. S. & Kaminski, C. F. (2013). *ChemBioChem*, **14**, 846–850. 10.1002/cbic.201300103 PMC3790954 23592254

[bb53] Poel, W. de, Tinnemans, P., Duchateau, A. L. L., Honing, M., Rutjes, F. P. J. T., Vlieg, E. & de Gelder, R. (2019). *Chem. Eur. J.* **25**, 14999–15003. 10.1002/chem.201904174 31529519

[bb54] Pravda, L., Sehnal, D., Toušek, D., Navrátilová, V., Bazgier, V., Berka, K., Svobodová Vařeková, R., Koča, J. & Otyepka, M. (2018). *Nucleic Acids Res.* **46**, W368–W373. 10.1093/nar/gky309 PMC6030847 29718451

[bb55] Qian, K., Liu, F., Yang, J., Huang, X., Gu, W., Jambhrunkar, S., Yuan, P. & Yu, C. (2013). *RSC Adv.* **3**, 14466.

[bb56] Rosenberger, L., von Essen, C., Khutia, A., Kühn, C., Urbahns, K., Georgi, K., Hartmann, R. W. & Badolo, L. (2020). *Drug Metab. Dispos.* **48**, 587–593. 10.1124/dmd.120.091140 32434832

[bb57] Sanna, E., Escudero-Adán, E. C., Bauzá, A., Ballester, P., Frontera, A., Rotger, C. & Costa, A. (2015). *Chem. Sci.* **6**, 5466–5472. 10.1039/c5sc01838b PMC5505123 28757946

[bb58] Schevitz, R. W., Otwinowski, Z., Joachimiak, A., Lawson, C. L. & Sigler, P. B. (1985). *Nature*, **317**, 782–786. 10.1038/317782a0 3903514

[bb59] Sehnal, D., Svobodová Vařeková, R., Berka, K., Pravda, L., Navrátilová, V., Banáš, P., Ionescu, C.-M., Otyepka, M. & Koča, J. (2013). *J. Cheminform*, **5**, 39. 10.1186/1758-2946-5-39 PMC3765717 23953065

[bb60] Shi, Y. (2014). *Cell*, **159**, 995–1014. 10.1016/j.cell.2014.10.051 25416941

[bb61] Shimanovich, U., Song, Y., Brujic, J., Shum, H. C. & Knowles, T. P. J. (2015). *Macromol. Biosci.* **15**, 501–508. 10.1002/mabi.201400366 25407891

[bb62] Smoluchowski, M. (1906). *Bull. Int. Acad. Sci. Cracovie*, pp. 577–602.

[bb63] Sprengel, A., Lill, P., Stegemann, P., Bravo-Rodriguez, K., Schöneweiss, E. C., Merdanovic, M., Gudnason, D., Aznauryan, M., Gamrad, L., Barcikowski, S., Sanchez-Garcia, E., Birkedal, V., Gatsogiannis, C., Ehrmann, M. & Saccà, B. (2017). *Nat. Commun.* **8**, 14472. 10.1038/ncomms14472 PMC5316895 28205515

[bb64] Squire, P. G. & Himmel, M. E. (1979). *Arch. Biochem. Biophys.* **196**, 165–177. 10.1016/0003-9861(79)90563-0 507801

[bb65] Tidow, H. & Nissen, P. (2013). *FEBS J.* **280**, 5551–5565. 10.1111/febs.12296 23601118

[bb66] Vagin, A. & Teplyakov, A. (2010). *Acta Cryst.* D**66**, 22–25. 10.1107/S0907444909042589 20057045

[bb67] Vallet-Regí, M., Colilla, M., Izquierdo-Barba, I. & Manzano, M. (2017). *Molecules*, **23**, 47. 10.3390/molecules23010047 PMC5943960 29295564

[bb68] Velev, O. D., Kaler, E. W. & Lenhoff, A. M. (2000). *J. Phys. Chem. B*, **104**, 9267–9275.

[bb69] Waltersson, Y., Linse, S., Brodin, P. & Grundstroem, T. (1993). *Biochemistry*, **32**, 7866–7871. 10.1021/bi00082a005 8347591

[bb70] Weichenberger, C. X. & Rupp, B. (2014). *Acta Cryst.* D**70**, 1579–1588. 10.1107/S1399004714005550 24914969

[bb71] Yan, E.-K., Cao, H.-L., Zhang, C.-Y., Lu, Q.-Q., Ye, Y.-J., He, J., Huang, L.-J. & Yin, D.-C. (2015). *RSC Adv.* **5**, 26163–26174.

[bb72] Yoshioka, S., Inokuma, Y., Hoshino, M., Sato, T. & Fujita, M. (2015). *Chem. Sci.* **6**, 3765–3768. 10.1039/c5sc01681a PMC5496191 28706719

