# Supplementary material for: Guest-protein incorporation into solvent channels of a protein host crystal (hostal)
Source: Acta Crystallogr D Struct Biol. 2021 Mar 30;77(Pt 4):471–85. doi: 10.1107/S2059798321001078 (PMC8025882; doi:10.1107/S2059798321001078)
Supplement: Supplementary file 1 [file d-77-00471-sup1.pdf]

STRUCTURAL
BIOLOGY

Volume 77 (2021)

Supporting information for article:

Guest protein incorporation into solvent channels of a protein host crystal (hostal)

Janina Sprenger, Jannette Carey, Alexander Schulz, Fleur Drouard, Catherine L. Lawson, Claes von Wachenfeldt, Sara Linse and Leila Lo Leggio

S1. Supplementary Material and Methods

S1.1. TrpR V58I expression and purification

Expression and purification of V58I TrpR with N–terminal 6x His-tag, spacer (SSGVDLGT) and rhinovirus 3C protease cleavage site (LEVLFQGP) was carried out as described by Sprenger *et al*. (2021, manuscript `Crystal structures of Val58Ile tryptophan repressor in domain-swapped array in presence and absence of L-tryptophan' submitted to *Acta Cryst. D*). Before crystallization, the purified TrpR (V58I) after cleavage of the His-tag was subjected to a final gel-filtration step (using a Superdex 75 300/10 column) with crystallization buffer (100 mM sodium phosphate buffer, 200 mM NaCl, pH 7.5). Fractions containing protein were analyzed for purity using SDS-PAGE and pure fractions were pooled and concentrated to 3 - 8.5 mg/ml (quantification by absorbance at 280 nm with extinction coefficient of 13980 M^{-1} cm^{-1}) using 3000 Da Amicon® Ultra-15 Centrifugal Filter Units.

S2. Supplementary Results

S2.1. Incorporation of Texas Red maleimide into the host crystal

To monitor diffusion of small molecules through the host crystal channels as comparison to later guest protein soaking trials, the fluorescent dye Texas Red maleimide was soaked into ds-TrpR host crystals. Host crystals were transferred into a drop of 4.5 µl containing 70 µM dye in reservoir solution on a microscope slide well. Because TrpR does not contain any cysteines, Texas Red maleimide can diffuse through the solvent channels but will not covalently attach to the protein. The diffusion of Texas Red was followed by CLSM using a time series in a focal plane through the center of the crystal (Fig. S3). The image series shows that the dye penetrates equally and rapidly through ~ 50 µm crystals. Most of the dye is absorbed by the crystals during the 5 min transfer time, but the staining is still slightly intensified over the next 20 min. The crystals furthermore enrich the dye significantly from the soaking solution by about 5-fold based on the fluorescence of the soaking stock compared to the fluorescence in the crystal.

S2.2. Estimation of guest content for soaking with high concentrations of CaM

The guest content estimation described here corresponds with the CaM soaking results described in the main text result section 3.3.3. The Alexa532-CaM fluorescence intensity within host crystals soaked for 2 weeks is 240 AU, which is above the detection range. This result suggests an enrichment of the guest within the crystals that was not observed at low concentrations of CaM. As the reservoir solution with 110 mg/ml CaM with 0.1% Alexa532-CaM gives a fluorescence of 60 AU (Fig. 5d) and background fluorescence of CaM without dye is neglectable, the fluorescence from the crystal corresponds to a CaM concentration > 400 mg/ml. Assuming this relationship between fluorescence and concentration is correct also in the crystal as no spectral change is observed, the minimum concentration of 400 mg/ml CaM in the crystal, as derived from to equation (2), results in:

$$\Phi_{guest} = \frac{400\ mg/ml}{1.37 \times 10^3\ mg/ml} = 0.29$$

This guest (v/v) % content in the crystal of ~ 30 % corresponds to ~ 40 % of the solvent channel occupied by guest protein. The molar ratio of CaM:TrpR is 1.1:1 in the crystal.

S2.3. Attempts to solve guest structures (molecular replacement)

The data set for the CytC-soaked crystal processed in $P6_122$ and in $P1$ used for the reported structure determinations was also used to attempt molecular replacement using the structure of CytC (PDB ID: 1HRC) as search model including ds-TrpR as fixed model, but no reasonable solution could be found. The same was the case with the data set for the CaM-soaked crystals using the calcium-free structure of CaM (PDB ID: 1CFD) or the separate N- or C-terminal domains from PDB 1CFD as search models. Although the guest structures could not be resolved, differences were observed in the data processing, structure refinement statistics, and channel electron density compared with the non-soaked structure. The raw diffraction data for soaking with both guests will be made publicly available to the community.

Table S1 Hydrodynamic properties calculation from HYDROPRO# (WinHydroPRO):

**Protein**	**PDB**	**MW (Da)**	**Rg (nm)**	**Dt (x 10^4 s^{-1})**	**Dmax (nm)**
Calmodulin (Ca2+-free)	1DMO *(NMR)*<br>1CFD *(NMR)*	16737	2.50<br>2.25	4.11<br>5.4	8.11<br>7.74
Calmodulin (Ca2+-bound)	1 EXR *(X-ray)*	16737	2.17	12.2	7.18
CytC, equus caballus	1HRC (*X-ray)*	11833	1.28	28.5	4.58

#Settings: Shell-model from atomic level, Specific volume 0.73 cm^3/g

Table S2 Fluorescence emission spectra at different excitation wavelength of soaked and unsoaked TrpR crystals and solutions including guest proteins.

**Method**	**Sample**	**λ_{ex} 355 nm (UV, CytC)**	**λ_{ex} 405 nm (UV,CytC)**	**λ_{ex} 528 nm (Green,Alexa532-CaM)**	**λ_{ex} 615 nm (Texas Red)**
**Microscope**		**SP5-X**	**SP5 inverted**	**SP5-X**	**SP5-X**
**CLSM, host crystal**	Non-soaked	420 - 600 nm<br>*peak: 465 nm*	430 - 600 nm<br>*broad peak 495 nm*	n.d.*	n.d.
	soaked with CytC	420 - 675 nm<br>*peak: 475, 510 nm with shoulder: 650 nm*	430 - 720 nm<br>*broad peak 505 nm, and shoulder/additional peak at 550 nm*	n.d.	n.d.
	soaked with Alexa532-CaM	420 - 660 nm<br>*peak: 550 nm and shoulder at 460 (TrpR crystal emission)*	-#	535 - 620<br>peak at 545 and shoulder at 560 nm	n.d.
**CLSM, solution**	TrpR 4.5 mg/ml	n.d.	n.d.	n.d.	n.d.
	CytC 150 mg/ml	n.d.	n.d.	n.d.	n.d.
	Alexa532-CaM 0.12 mg/ml	515 - 660 nm, peak at 550 nm	-	535 - 620<br>peak at 545 and shoulder at 560 nm	n.d.

* 'n.d.' = not detected

'-'= not measured

Table S3 Cell- parameters (a) and processing statistics (b) from XDS for ds-TrpR host crystals without guest.

(a)

**Crystal ID**	**Cell dimensions**			
	***P*1**		***P*6$_1$22**	
	**a,b,c (Å)**	**α,β,γ (°)**	**a,b,c (Å)**	**α,β,γ (°)**
**Hostal_1**	85.5 85.6 115.1	90.2 90.1 120.3	86.4 86.4 115.8	
**Hostal_2**	85.4 85.6 114.9	90.0 90.0 119.8	85.5 85.5 114.9	
**Hostal_3**	85.3 85.4 114.9	90.1 90.0 119.9	85.3 85.3 114.9	90.0 90.0 120.0
**Hostal_4**	85.4 85.6 114.9	90.0 90.0 119.9	85.5 85.5 114.9	

(b)

**Crystal ID**	***P*1**					***P*6$_1$22**				
	Res., high (Å)	$CC_{1/2}$	I/σ	Complet. (%)	R_{meas} (%)	Res. high (Å)	$CC_{1/2}$	I/σ	Complet. (%)	R_{meas} (%)
**Hostal_1**	3.25	99.5 (53.8)[#]	8.37 (1.40)	98.8 (98.7)	10.3 (100.3)	2.90	99.9 (47.5)	18.34 (0.97)	100.0 (100.0)	13.2 (389.5)
**Hostal_2**	2.65	99.9 (61.2)	11.45 (1.19)	98.5 (98.6)	7.2 (108.4)	2.40	99.9 (66.9)	25.52 (1.29)	100.0 (100.0)	9.1 (263.7)
**Hostal_3**	3.30	99.3 (52.5)	6.71 (1.28)	98.9 (98.7)	12.4 (105.9)	3.00	99.8 (60.6)	14.27 (1.31)	100.0 (100.0)	15.8 (337.2)
**Hostal_4**	2.55	99.8 (52.3)	6.88 (1.16)	89.6 (91.1)	8.6 (86.8)	2.20	99.9 (47.0)	18.37 (0.63)	100.0 (99.9)	11.0 (456.6)

[#]Numbers in brackets represent values for the highest resolution shell.

Table S4 Cell- parameters (a) and processing statistics (b) from XDS for ds-TrpR crystals after ~ 2 weeks soaking with CaM.

(a)

Crystal ID	**Cell dimensions**			
	***P*1**		***P*6$_1$22**	
	**a,b,c (Å)**	**α,β,γ (°)**	**a,b,c (Å)**	**α,β,γ (°)**
**CaM_1**	85.7 85.6 115.1	90.01 90.08 119.9	85.6 85.6 115.1	90.0 90.0 120.0
**CaM_2**	84.8 85.0 114.2	90.1 90.0 120	84.9 84.9 114.2	
**CaM_3**	84.8 85.0 114.6	90.0 90.0 120	84.9 84.9 114.2	
**CaM_4**	84.8 85.0 114.6	90.0 90.0 119.8	84.9 84.9 114.6	

(b)

**Crystal ID**	***P*1**					***P*6$_1$22**				
	Res., high (Å)	$CC_{1/2}$	I/σ	Complet. (%)	R_{meas} (%)	Res. high (Å)	$CC_{1/2}$	I/σ	Complet. (%)	R_{meas} (%)
**CaM_1**	3.40	99.8 (62.4)[#]	18.23 (1.39)	90.4 (91.0)	8.8 (73.2)	2.90	100.0 (57.6)	14.50 (0.92)	99.9 (100.0)	14.5 (379.0)
**CaM_2**	3.20	98.6 (53.6)	5.43 (1.24)	98.4 (98.7)	18.1 (102.0)	2.80	99.8 (55.9)	11.68 (1.05)	99.9 (100.0)	23.9 (353.6)
**CaM_3**	2.85	99.4 (47.7)	6.93 (1.02)	98.8 (98.4)	13.6 (127.5)	2.45	99.9 (44.3)	15.01 (0.89)	100.0 (100.0)	18.6 (357.1)
**CaM_4**	3.05	99.5 (48.8)	7.08 (1.11)	96.9 (95.7)	13.9 (109.9)	2.75	99.9 (50.9)	15.87 (1.04)	100.0 (100.0)	18.1 (278.8)

[#]Numbers in brackets represent values for the highest resolution shell.

Table S5 Cell- parameters (a) and processing statistics (b) from XDS for ds-TrpR crystals after ~ 2 weeks soaking with CytC.

(a)

Crystal ID	Cell dimensions			
	*P*1		*P*$6_1$22	
	a,b,c (Å)	α,β,γ (°)	a,b,c (Å)	α,β,γ (°)
**Cyt_1**	85.2 85.3 115.2	90.0 90.0 120.0	85.3 85.3 115.2	90.0 90.0 120.0
**Cyt_2**	85.5 85.6 115.0	90.0 90.0 120.0	85.6 85.6 115.0	
**Cyt_3**	85.5 85.5 115.0	90.0 90.0 119.9	85.5 85.5 115.0	
**Cyt_4**	85.3 85.3 115.1	90.0 90.0 120.0	85.3 85.3 115.1	

(b)

**Crystal ID**	*P*1					*P*$6_1$22				
	Res., high (Å)	$CC_{1/2}$	I/σ	Complet. (%)	R_{meas} (%)	Res. high (Å)	$CC_{1/2}$	I/σ	Complet. (%)	R_{meas} (%)
**Cyt_1**	3.10	99.8 (60.9)#	8.15 (1.24)	98.8 (98.6)	12.0 (121.1)	2.75	100.0 (71.9)	18.78 (1.32)	99.9 (99.6)	16.2 (307.5)
**Cyt_2**	2.85	99.8 (66.9)	8.61 (1.38)	98.9 (98.7)	11.5 (103.2)	2.80	100.0 (61.5)	118.08 (0.97)	100.0 (99.8)	19.9 (532.3)
**Cyt_3**	3.37	99.9 (58.5)	8.26 (0.96)	98.8 (98.5)	13.5 (163.9)	2.95	99.9 (47.1)	16.85 (0.62)	99.9 (100.0)	21.5 (747.6)
**Cyt_4**	3.40	99.8 (52.0)	7.82 (1.21)	98.9 (98.9)	14.3 (125.9)	2.95	100.0 (54.8)	19.33 (1.10)	99.9 (99.7)	20.3 (432.0)

#Numbers in brackets represent values for the highest resolution shell.

Table S6 Variations in cell dimension and merging statistics between data sets after processing in *P*1 and *P*$6_1$22 (a)

**Crystal (ds-TrpR)**	**Variations between data sets***								
	***P*1**					***P*$6_1$22**			
	**Number of sets**	**a (Å)**	**b (Å)**	**c (Å)**	**R_{meas} (%)**	**Number of sets**	**a,b (Å)**	**c (Å)**	**R_{meas} (%)**
**Non-soaked**	4	85.4* +/- 0.1	85.6 +/- 0.1	115.0 +/- 0.1	9.65 +/- 2.3	4	85.8 +/- 0.5	114.5 +/- 0.4	12.3 +/-2.9
**CaM soaked**	4	85.0 +/- 0.5	85.15 +/- 0.25	114.7 +/- 0.25	13.6 +/- 3.8	4	85.1 +/- 0.3	114.5 +/- 0.4	18.6 +/- 3.9
**CytC soaked**	4	85.4 +/- 0.15	85.4 +/-0.15	115.1 +/- 0.1	12.8 +/- 1.3	4	85.4 +/- 0.2	115 +/- 0.1	19.5 +/- 2.3

*High resolution cut-off based on the criteria $CC_{1/2} > 0.5$ in the highest resolution shell after first processing of the full resolution range of the collected data in *P*1 or *P*6122, respectively with XDS

Table S7 Data collection and refinement statistics for TrpR (V58I) as host crystal without soaked guest.[*]

**TrpR (V58I) data** ID: Hostal_2	**Highest res. *P*1**	**4Å, *P*1**	**Highest res. *P* 6_1 2 2**	**4Å, *P* 6_1 2 2**
**Data collection and refinement statistics**				
Wavelength (Å)	0.967689			
Data collection source	MAXIV, BioMAX beamline			
Resolution range (Å)	45.34 - 2.5 (2.59 - 2.50)[#]	45.34 - 4.0 (4.14 - 4.00)	42.72 - 2.40 (2.48 - 2.40)	42.72 - 4.0 (4.14 - 4.00)
Space group	*P* 1	*P* 1	*P* 6_1 2 2	*P* 6_1 2 2
Unit cell: a,b,c (Å); α,β,γ (°)	85.46 85.42 114.69 90.04 90.01 112.00	85.46 85.42 114.69 90.04 90.01 112.00	85.45 85.45 114.71 90 90 120	85.45 85.45 114.71 90 90 120
Total reflections	384747 (39378)	94947 (9331)	437203 (43294)	95073 (9209)
Unique reflections	95670 (9571)	23451 (2274)	10268 (997)	2348 (215)
Multiplicity	4.0 (4.1)	4.0 (4.1)	42.6 (43.4)	40.5 (42.6)
Completeness (%)	98.33 (97.80)	98.57 (99.09)	99.89 (99.80)	99.83 (100.00)
$I/\sigma(I)\rangle$	10.08 (0.78)	26.13 (19.95)	26.33 (1.27)	72.37 (57.22)
Wilson B-factor (A2)	71.89	70.22	71.87	77.41
R_{merge} (%)	6.95 (131.8)	3.91 (59.65)	9.23 (245.7)	5.04 (78.00)
R_{meas} (%)	8.04 (151.8)	4.512 (68.65)	9.34 (248.6)	5.11 (78.93)
$CC_{1/2}$	0.999 (0.336)	0.999 (0.995)	0.999 (0.712)	0.999 (0.999)
CC*	1.000 (0.709)	1.000 (0.999)	1.000 (0.912)	1.000 (1.000)
**Refinement statistics**				
R-work (%)	26.85 (41.35)	19.51 (25.70)	26.23 (38.67)	18.91 (22.55)
R-free (%)	31.01 (43.30)	24.38 (31.93)	29.92 (34.54)	24.69 (28.12)
No. of macromolecules	12	12	1	1
No. of solvent molecules	-	-	24	24
r.m.s. deviations				
Bonds (Å)	0.004	0.002	0.008	0.003
Angles (°)	0.77	0.49	1.12	0.63
Ramachandran favored (%)	96.41	97.39	97.06	97.06
Ramachandran allowed (%)	3.43	2.61	2.94	2.94
Ramachandran outliers (%)	0.16	0.00	0.00	0.00
Rotamer outliers (%)	0.19	0.09	0.00	0.00
Clashscore	6.38	3.41	9.68	2.28
Average overall B-factor	84.01	72.43	96.71	77.99

[*]This table is generated with phenix.table_one and processing statistics may deviate (due to different range of highest-resolution shell) from data presented in tables S3-S5 that report XDS output.

[#]Statistics for the highest-resolution shell are shown in parentheses.

Table S8 Data collection and refinement statistics for host crystal with incorporated CaM.

**CaM soaked**<br>ID: CaM_3	**Highest res. *P*1**	**4Å, *P*1**	**Highest res. *P* 6_1 2 2**	**4Å, *P* 6_1 2 2**
**Data collection and refinement statistics**				
Wavelength (Å)	0.967689			
Data collection source	MAXIV, BioMAX beamline			
Resolution range (Å)	45.09 - 2.85<br>(2.95 - 2.85)[)#]	45.09 - 4.0<br>(4.14 - 4.0)	45.22 - 2.452<br>(2.54 - 2.45)	45.22 - 4.00<br>(4.15 - 4.00)
Space group	*P* 1	*P* 1	*P* 6_1 2 2	*P* 6_1 2 2
Unit cell: a,b,c (Å);<br>α,β,γ (°)	84.69 84.95 114.23<br>90.13 89.91 119.89	84.69 84.95 114.23<br>90.13 89.91 119.89	85.10 85.10 114.55<br>90 90 120	85.10 85.10 114.55<br>90 90 120
Total reflections	254403 (25277)	93399 (9440)	402177 (38117)	93991 (9429)
Unique reflections	63664 (6309)	23103 (2302)	9483 (901)	2328 (217)
Multiplicity	4.0 (4.0)	4.0 (4.1)	42.4 (42.3)	40.4 (43.3)
Completeness (%)	98.64 (98.38)	98.76 (99.14)	99.80 (98.79)	99.87 (100.00)
$I/\sigma(I)\rangle$	7.23 (1.01)	13.46 (10.83)	15.02 (0.75)	40.18 (33.28)
Wilson B-factor (A^2)	79.54	69.02	74.49	75.19
R_{merge} (%)	11.99 (105.9)	81.57 (11.04)	18.37 (417)	9.837 (13.8)
R_{meas} (%)	13.84 (122.2)	9.44 (12.75)	18.59 (422)	9.96 (13.97)
$CC_{1/2}$	0.994 (0.499)	0.994 (0.983)	0.999 (0.314)	0.999 (0.998)
CC*	0.998 (0.816)	0.998 (0.996)	1.000 (0.692)	1.000 (1.000)
**Refinement statistics**				
R-work (%)	25.55 (34.35)	22.56 (28.72)	28.24 (37.74)	25.69 (27.48)
R-free (%)	30.76 (38.93)	27.18 (33.31)	32.87 (45.75)	28.75 (36.62)
No. of macromolecules	12	12	1	1
No. of solvent molecules	-	-	24	24
r.m.s. deviations				
Bonds (Å)	0.007	0.002	0.002	0.001
Angles (°)	0.90	0.41	0.43	0.34
Ramachandran favored (%)	96.65	99.18	94.12	100.00
Ramachandran allowed (%)	3.10	0.82	5.88	0.00
Ramachandran outliers (%)	0.25	0.00	0.00	0.00
Rotamer outliers (%)	0.00	0.00	0.00	0.00
Clashscore	8.71	3.07	2.87	2.29
Average overall B-factor	84.65	66.70	95.70	91.18

[*]This table is generated with phenix.table_one and processing statistics may deviate (due to different range of highest-resolution shell) from data presented in tables S3-S5 that report XDS output.

[#]Statistics for the highest-resolution shell are shown in parentheses.

Table S9 Data collection and refinement statistics for host crystal with incorporated CytC.

**CytC soaked**<br>ID: Cyt_1	**Highest res. *P*1**	**4Å, *P*1**	**Highest res. *P* 6_1 2 2**	**4Å, *P* 6_1 2 2**
**Data collection and refinement statistics**				
Wavelength (Å)	0.967689			
Data collection source	MAXIV, BioMAX beamline			
Resolution range (Å)	45.38 - 2.85 (2.95 - 2.85)[#]	45.38 - 4.00 (4.14 - 4.00)	42.62 - 2.75 (2.85 - 2.75)	42.62 - 4.0 (4.14 - 4.0)
Space group	*P* 1	*P* 1	*P* 6_1 2 2	*P* 6_1 2 2
Unit cell: a,b,c (Å); α,β,γ (°)	85.18 85.21 115.09 90.01 89.99 120.02	85.18 85.21 115.09 90.01 89.99 120.02	85.23 85.23 115.15 90 90 120	85.23 85.23 115.15 90 90 120
Total reflections	256900 (25320)	94749 (9497)	286552 (27502)	95033 (9417)
Unique reflections	64530 (6318)	23449 (2324)	6880 (659)	2347 (219)
Multiplicity	4.0 (4.0)	4.0 (4.1)	41.6 (41.7)	40.5 (43.0)
Completeness (%)	98.39 (97.87)	98.56 (99.15)	99.69 (99.24)	99.74 (100.00)
$I/\sigma(I)\rangle$	6.72 (0.57)	14.46 (8.47)	19.40 (1.23)	44.46 (27.00)
Wilson B-factor (A^2)	82.54	85.84	80.85	91.92
R_{merge} (%)	12.71 (194.70)	6.46 (14.50)	16.24 (308.40)	7.62 (17.0)
R_{meas} (%)	14.72 (225.40)	7.47 (16.75)	16.44 (312.10)	7.72 (17.2)
$CC_{1/2}$	0.997 (0.336)	0.998 (0.984)	0.999 (0.708)	1.000 (0.999)
CC*	0.999 (0.709)	0.999 (0.996)	1.000 (0.911)	1.000 (1.000)
**Refinement statistics**				
R-work (%)	26.20 (40.13)	19.53 (23.45)	31.19 (41.59)	25.94 (28.61)
R-free (%)	30.20 (41.02)	24.00 (30.10)	37.63 (56.37)	31.82 (25.10)
Number of macromolecules	12	12	1	1
Number of solvent	-	-	24	24
r.m.s. deviations				
Bonds (Å)	0.017	0.003	0.013	0.001
Angles (°)	0.81	0.55	0.83	0.40
Ramachandran favored (%)	96.24	97.79	100.00	100.00
Ramachandran allowed (%)	3.43	2.04	0.00	0.00
Ramachandran outliers (%)	0.33	0.16	0.00	0.00
Rotamer outliers (%)	0.00	0.00	0.00	0.00
Clashscore	13.51	3.61	8.08	4.04
Average overall B-factor	91.05	91.23	86.59	98.89

[*]This table is generated with phenix.table_one and processing statistics may deviate (due to different range of highest-resolution shell) from data presented in tables S3-S5 that report XDS output.

[#]Statistics for the highest-resolution shell are shown in parentheses.

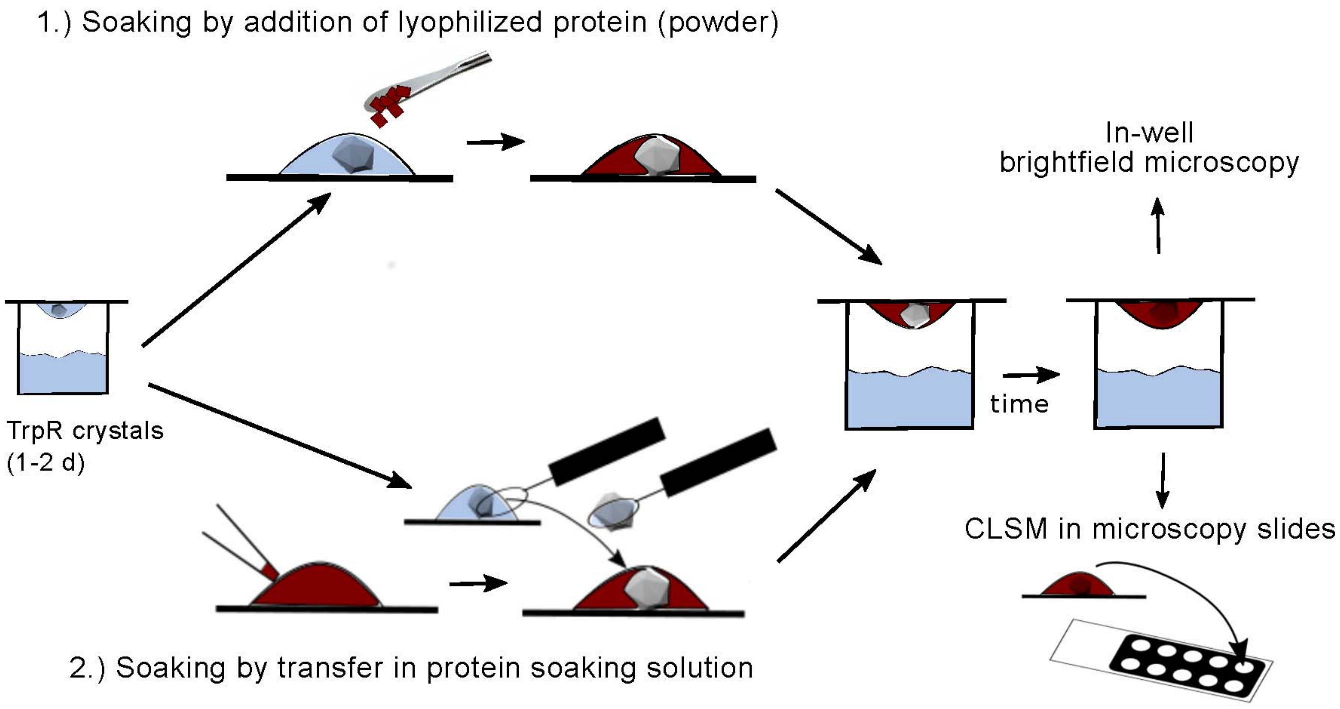

Figure S1 Soaking procedures for guest protein incorporation and imaging. Crystals grown in hanging drops were soaked in two different ways. 1) For soaking without controlling the concentration of the guest protein, the guest was added as lyophilized powder directly to the crystal drop on the cover slip that was transferred back to the hanging drop set-up to prevent evaporation (not shown). 2) Soaking stock solution with controlled guest protein concentration was added to a cover slip and crystals were transferred into the guest-protein drop using crystal loops. The cover slip was back-transferred to the hanging drop set-up to prevent evaporation (not shown). Imaging of the crystals using a brightfield microscope was done on drops in their well setups. For imaging using CLSM the crystals were transferred either by pipette or crystal loops into the well of a microscope slide pre-filled with reservoir or soaking solution and covered by a cover slip.

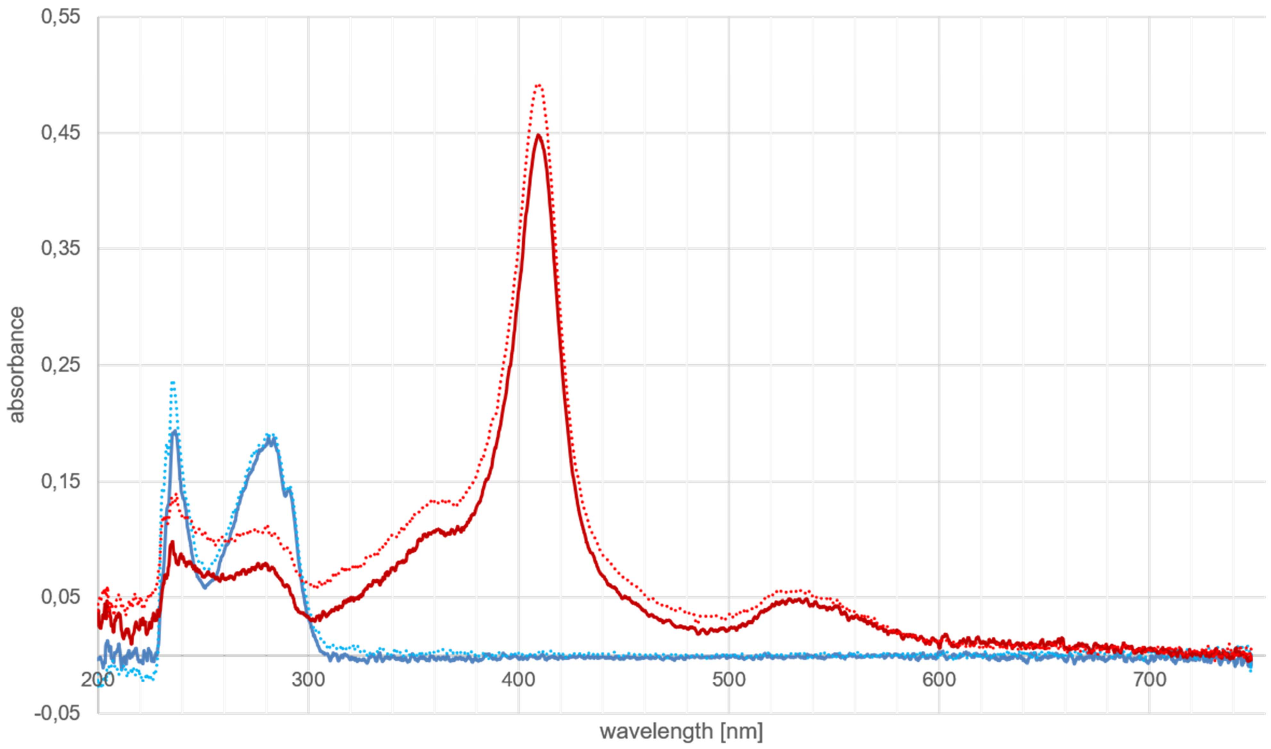

Figure S2 Absorbance spectra of CytC and TrpR.Absorbance spectra were recorded on a Probedrum spectrophotometer (Probation Labs Sweden AB) for 12 µM TrpR (blue) and 4 µM CytC (red) in 100 mM HEPES, 100 NaCl, pH 7.5 (solid lines) and in the same buffer with addition of isopropanol to 16 % final concentration (dotted lines).

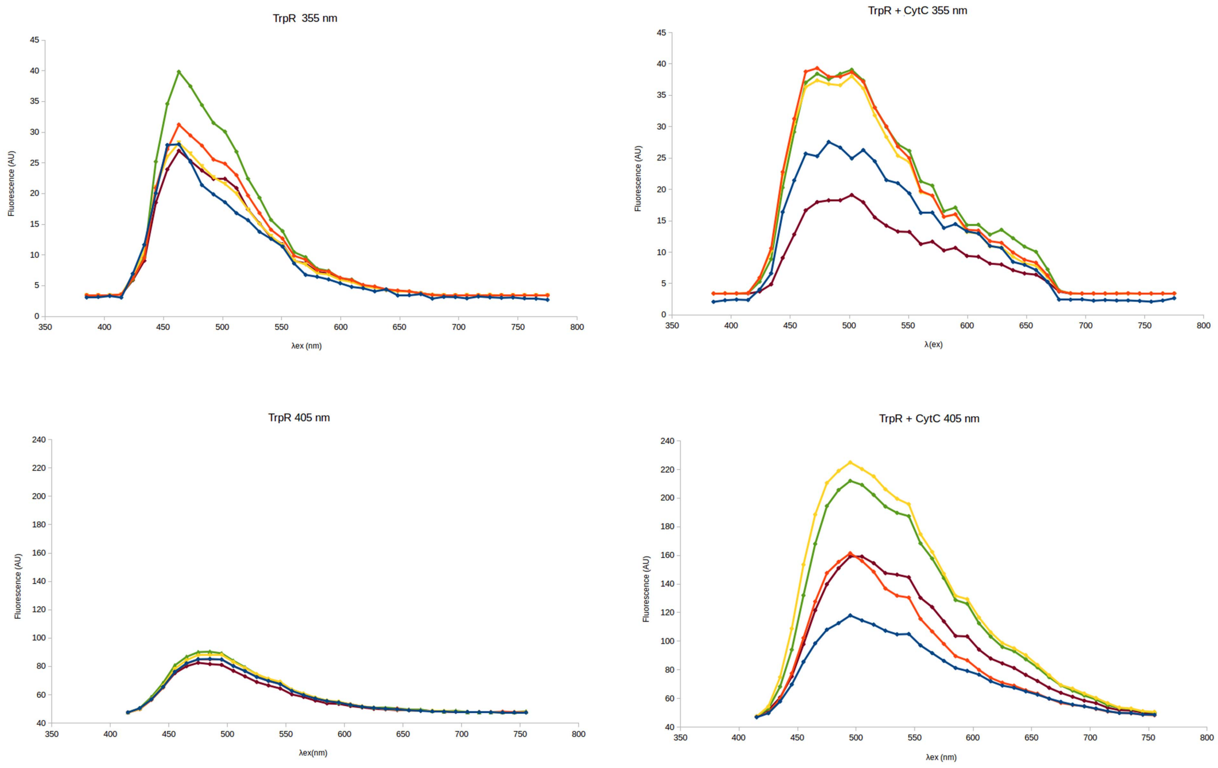
TrpR 355 nm
Fluorescence (AU)
λex (nm)
TrpR + CytC 355 nm
Fluorescence (AU)
λ(ex)
TrpR 405 nm
Fluorescence (AU)
λex(nm)
TrpR + CytC 405 nm
Fluorescence (AU)
λex (nm)

(a)

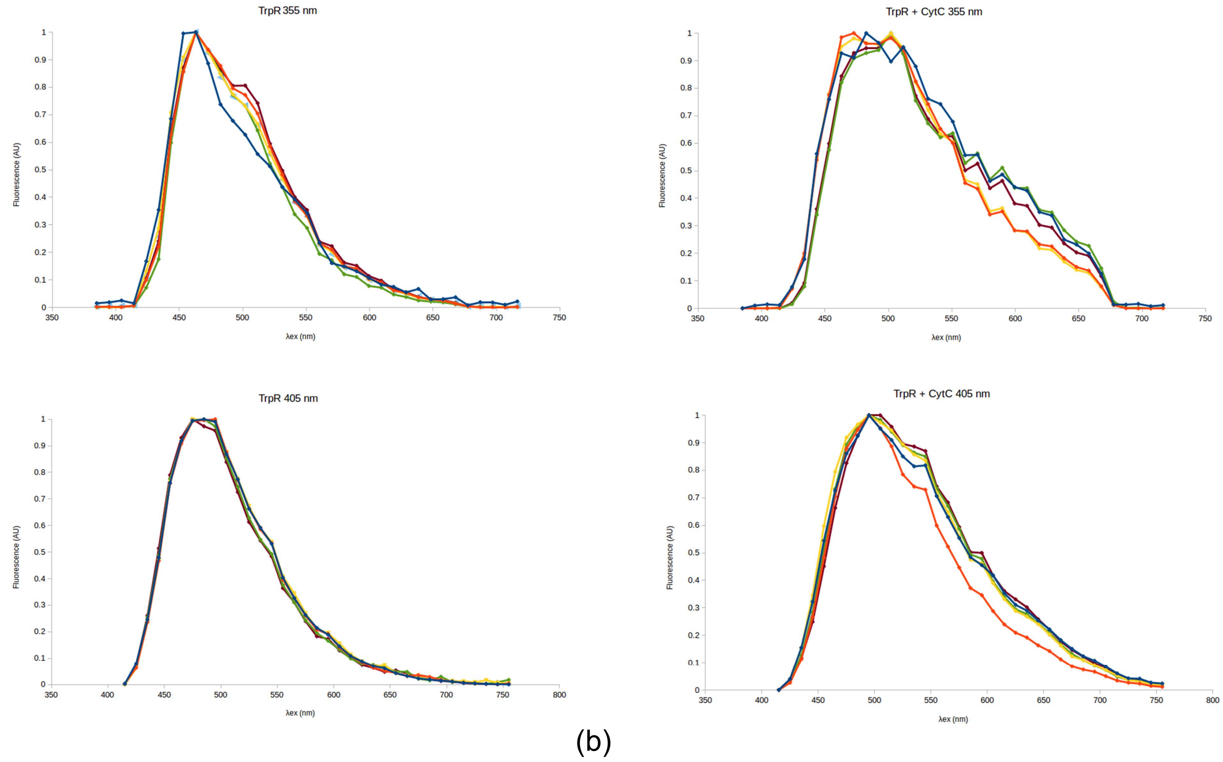
TrpR 355 nm
Fluorescence (AU)
λex (nm)
TrpR + CytC 355 nm
Fluorescence (AU)
λex (nm)
TrpR 405 nm
Fluorescence (AU)
λex (nm)
TrpR + CytC 405 nm
Fluorescence (AU)
λex (nm)

(b)

Figure S3 Fluorescence emission spectra of ds-TrpR crystals. (a) Emission spectra with fluorescence intensities and (b) normalized intensities obtained as explained in the experimental part of the main text and Figure 5. Each differently colored line with dots represents a spectrum of an individual crystal of the ds-TrpR host (left)or CytC-soaked ds-TrpR (right) at the excitation wavelength indicated.

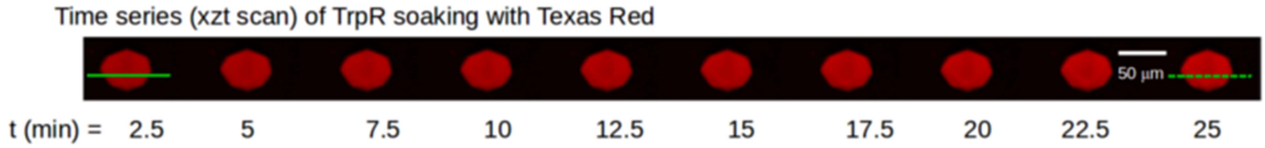

(a)

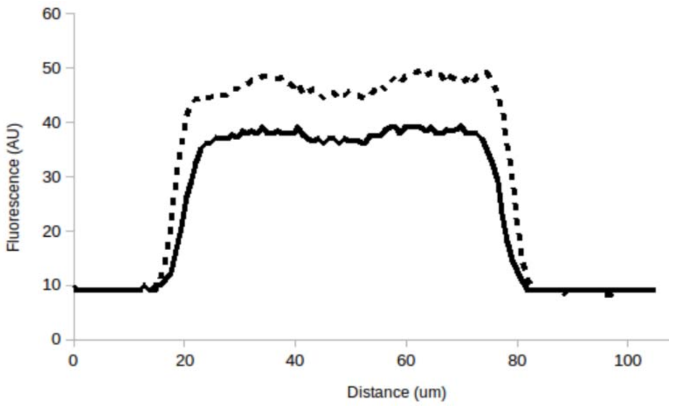

(b)

Figure S4 Texas Red soaking. (a) Time series (xyt-scan) with focal plane in the center of a 45 µm ds-TrpR host crystal following soaking with 70 µM Texas Red maleimide dye in reservoir solution over 25 min. The excitation wavelength was 594 nm and the fluorescence emission was detected between 615-674 nm. The fluorescence emission of the Texas Red in the crystal intensifies only slightly over the 25 min from ~ 40 to 50 ~ AU and is significantly above the intensity of the surrounding Texas Red in the surrounding reservoir (9 AU). (b) Fluorescence intensity along the indicated green lines through the crystals in (a) after 2.5 min (solid line) and 25 min (dotted line) of soaking.

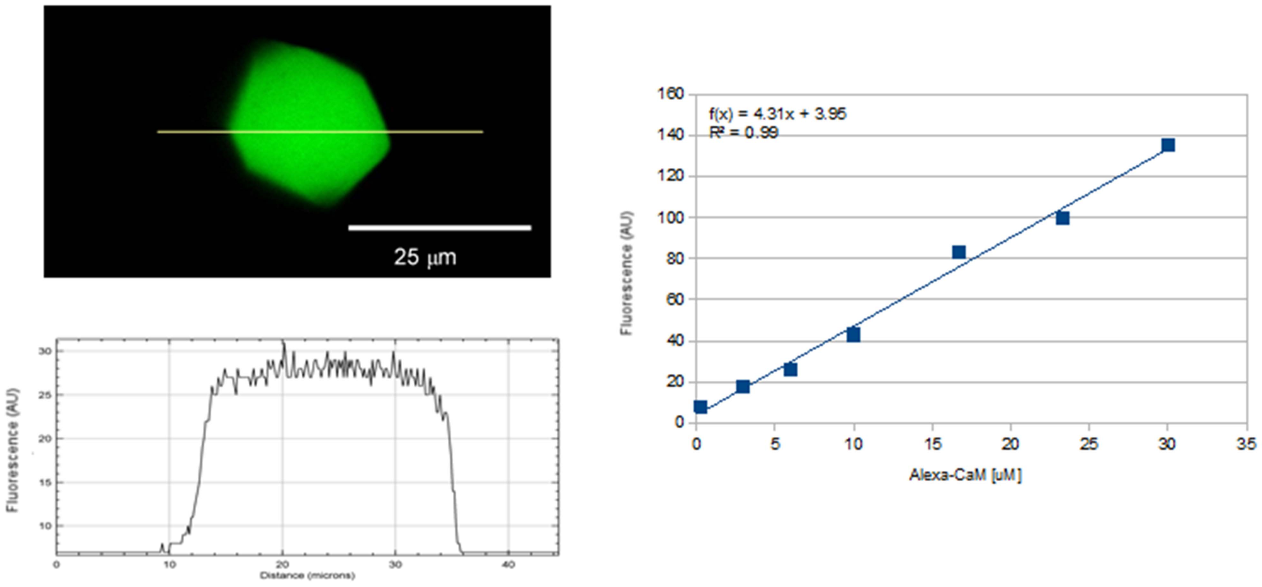

Figure S5 Quantification of Alexa532-CaM soaked ds-TrpR crystals. Microscope image and intensity plot for 15 µm Alexa532-CaM soak for 1 week and Alexa532-CaM standard. Fluorescence microscopy image (excitation at 528 nm and emission at 550 - 589 nm) at focal plane through center of a TrpR crystal (~ 20 µm) soaked for 1 week with 15 µM Alexa532-CaM. The fluorescence intensity along the indicated line through the crystal is shown as plot below. For quantification the Alexa532-CaM fluorescence is compared to the standard curve of Alexa532-CaM solutions using the same CLSM settings (right).

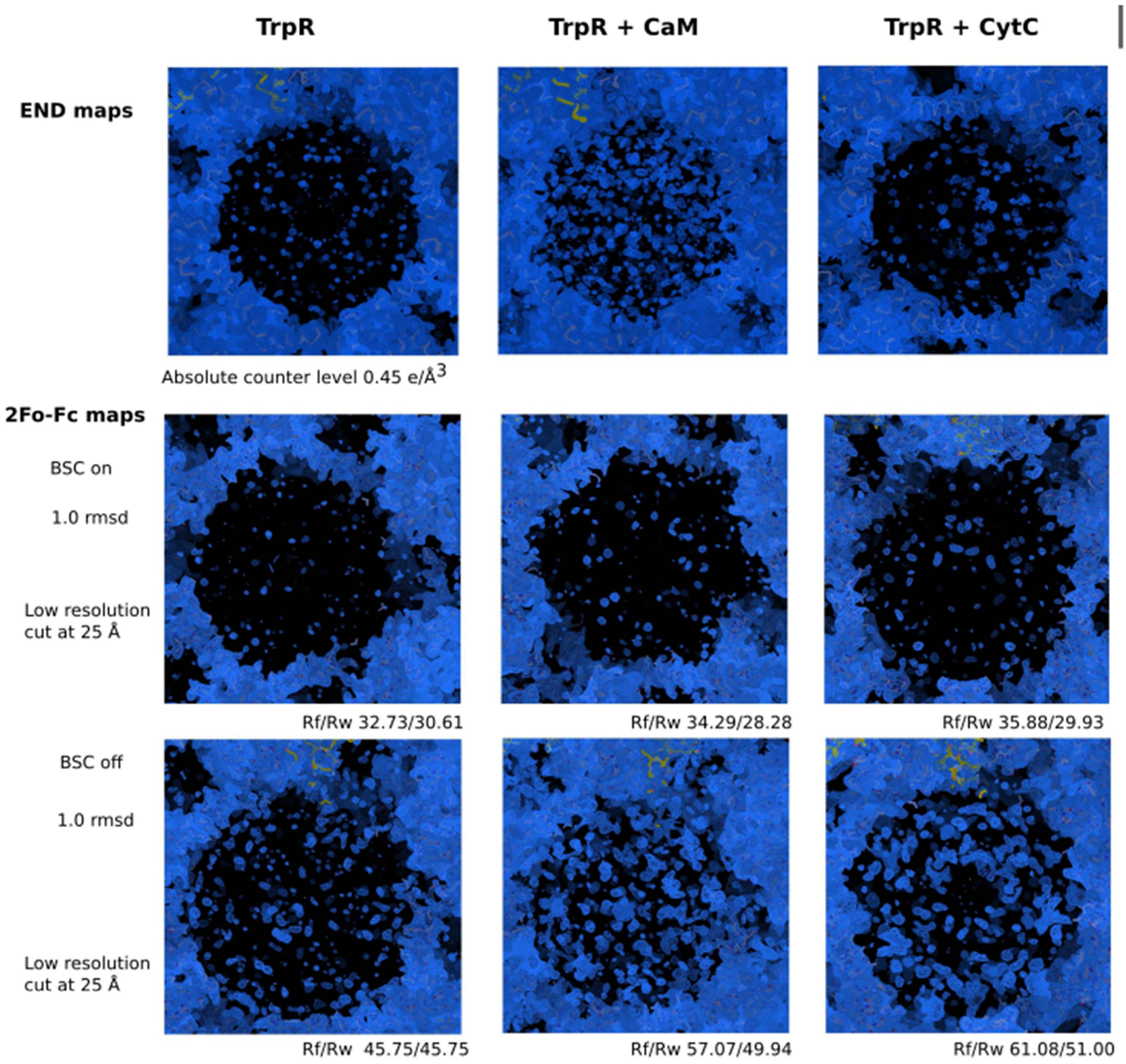

Figure S6 END maps and 2Fo-Fc +/- bulk solvent correction maps. END maps (upper three panels) were calculated as described in Methods for the indicated data sets (full processing statistics in SI, Tables S7-9) and the channel density is presented at an absolute contour level of 0.45 electrons per unit area ($e^-/Å^3$). 2Fo-Fc maps calculated using phenix.refine with a low-resolution cut-off at 25 Å are shown in the middle three panels including bulk solvent correction (BSC) and for the lower three panels without BSC.

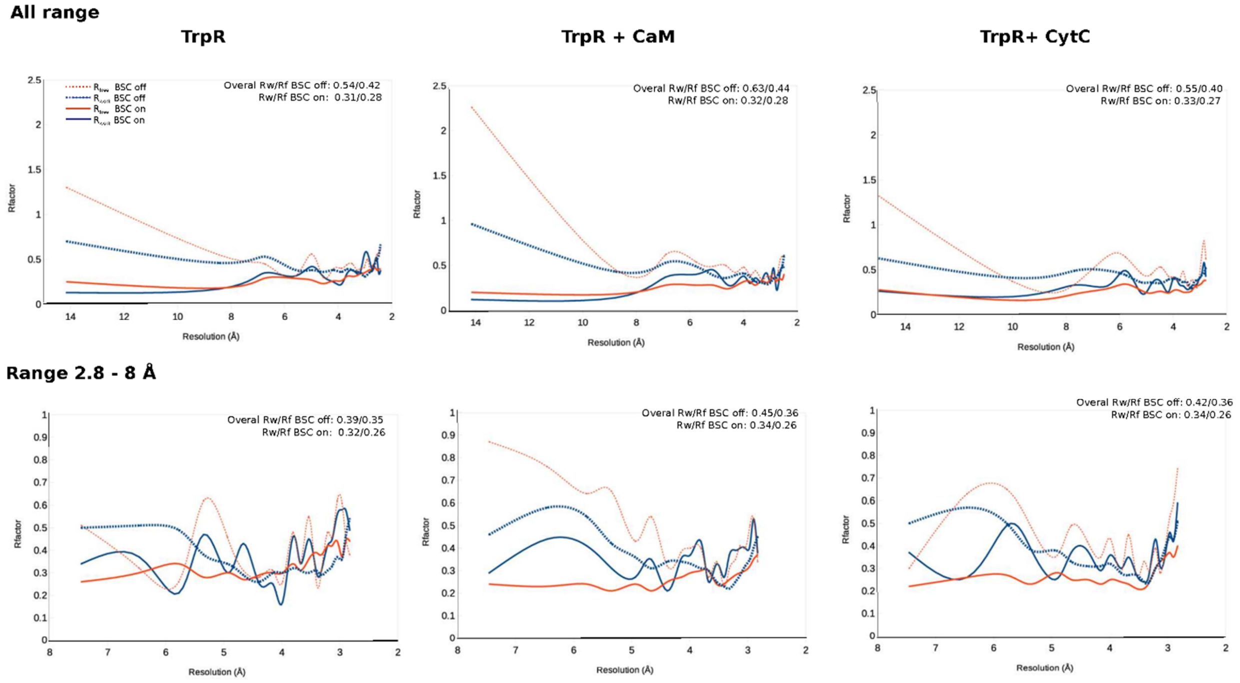

Figure S7 Resolution-dependent effect of bulk solvent correction on refinement parameters. R-factors (R_{free} in red and R_{work} in blue) are presented as function of resolution for the indicated data sets after 30 cycles of restrained refinement with refmac in space group $P6_122$. The refinement was done including either the entire resolution range (upper row; data sets reported in SI, Tables S7-9) or including only reflections between 2.8 – 8 Å (lower row). Bulk solvent correction (BSC) was applied as indicated by the legend in the upper left panel. The overall R_{work}/R_{free} values are given in each panel.

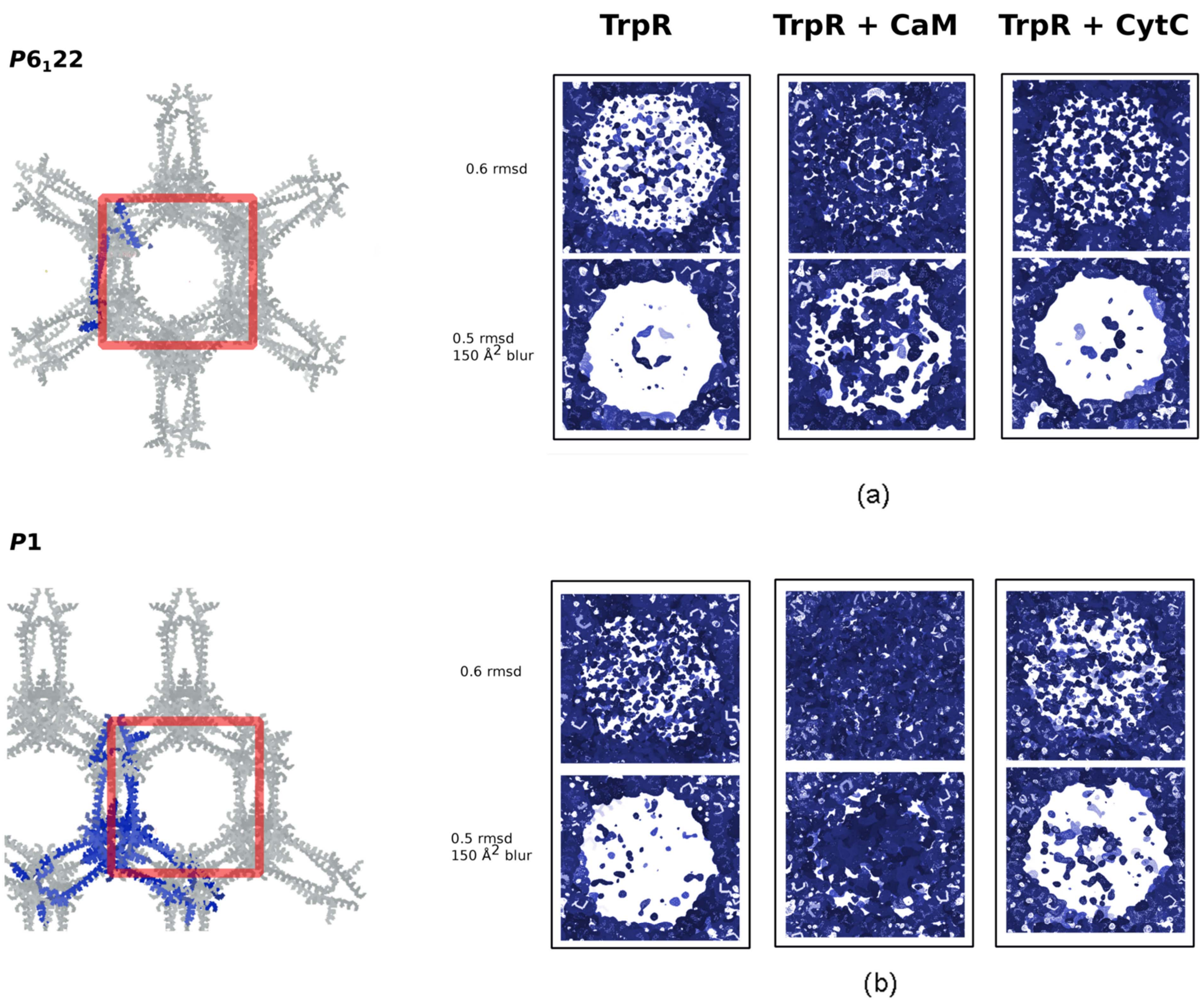

Figure S8 Guest electron density in solvent channels for 4 Å truncated data. Crystals like those in Figure 6d after 15 days of soaking with Alex-CaM or CytC were diffracted at BioMAX, Lund. The data sets were processed in *P*6122 (a) or *P*1 (b). Left, protein assembly cartoons (gray ribbons) were generated from the molecule(s) (blue ribbon) in the asymmetric unit surrounding each channel area marked with a red square. Right, electron density within the channel areas corresponding to each red square on the left for ds-TrpR crystals alone or soaked with Alexa-CaM or CytC as indicated. Each structure was solved at 4 Å resolution. Electron density is represented as 2Fo-Fc maps at 0.6 rmsd contour level (upper panels), or at 0.5 rmsd contour level with blurring to 150 A^2 (lower panels). The images were generated using Coot.
